# Closed-loop cognitive state control: a hypothesis framework for real-time estimation, stabilization, and recovery of task-specific high-performance cognitive states

**DOI:** 10.3389/fnrgo.2026.1909443

**Published:** 2026-10-27

**Authors:** Ibrahim Mourad

**Affiliations:** Paragon Intelligence LLC, Chantilly, VA, United States

**Keywords:** closed-loop control, cognitive state estimation, digital biomarkers, digital twin, human performance, neuroadaptive technology, neuroengineering, neuroergonomics

## Abstract

Human performance often fails not because latent capacity is absent, but because access to high-functioning cognitive states is unstable under fatigue, distraction, overload, and pressure. This hypothesis paper proposes *closed-loop* cognitive state control—informally, *supercognition*—as a neuroengineering objective: rapid entry into, stabilization of, and recovery of an individual's highest available task-specific cognitive state. The framework treats cognition as a partially observable dynamical system whose latent states would be inferred from multimodal behavioral, physiological, contextual, and neural signals, and specifies a five-layer architecture comprising multimodal sensing, latent-state inference, predictive transition modeling, closed-loop intervention, and a longitudinal personal neural twin. The proposal is situated against existing closed-loop and neuroadaptive architectures, with an explicit statement of what is and is not new. The state ontology is given as a set of falsifiable assumptions rather than an intuitive taxonomy. Candidate computational implementations are specified for each layer, including hidden semi-Markov and switching state-space models, Bayesian and Kalman filtering, sequence models, hazard models, contextual bandits, model-predictive control, and offline reinforcement learning. The personal neural twin receives an operational specification covering its data requirements, update schedule, calibration and drift-monitoring criteria, and failure modes. Predictive and causal claims are separated, with the evidentiary requirements stated for each. The paper engages directly with the literature that challenges the framework's feasibility, including biomarker reliability, mixed neurofeedback findings, cross-task and cross-subject generalization failure, and the ecological-validity gap in wearable neurotechnology. Operational definitions, application scenarios, a validation protocol with power and diversity requirements, an expanded ethics and governance analysis, and a staged research roadmap are provided. No component of the architecture has been implemented or evaluated here: no simulation, clinical trial, or empirical efficacy claim is presented, and the validation programme described is prospective. The contribution is a falsifiable systems-engineering hypothesis for cognitive-state control.

## 1 Introduction

Cognitive enhancement is often described as making people more intelligent. That framing is too broad to guide engineering. In many real tasks, performance failure is not caused by the absence of latent ability; it is caused by unstable access to the cognitive state in which that ability is available. A person may reason well in one interval and poorly in the next without any meaningful change in trait intelligence. Fatigue, stress, interruption, affective interference, poor arousal regulation, and attentional drift move the operating point of the cognitive system (Warm et al., 2008; Lim and Dinges, 2008; Dehais et al., 2020).

This paper therefore reframes cognitive enhancement as a closed-loop control problem. The target is not permanent trait transformation but reliable entry into, maintenance of, and recovery of high-performing states already feasible for the individual. The user does not need a new mind. The user needs better real-time control over the best functional state that the existing nervous system can safely express.

The reframing is consistent with established directions in neurotechnology. Brain–computer interfaces (BCI) convert neural signals into control or communication channels (Wolpaw et al., 2002; Lotte et al., 2018). Passive BCIs estimate user states such as workload, attention, fatigue, or engagement without volitional control (Zander and Kothe, 2011; Zander et al., 2016; Krol et al., 2020). Neurofeedback uses measured brain activity to train or regulate neural dynamics (Sitaram et al., 2017; Ros et al., 2020; Thibault et al., 2016). Adaptive automation and closed-loop stimulation use state estimates to select interventions in real time (Oehrn et al., 2024; Little et al., 2013; Morrell, 2011; Wilson and Russell, 2007; Aricò et al., 2016). The framework proposed here integrates these lines under a single objective: infer the user's current cognitive regime, predict adverse transitions, intervene before performance collapse, and learn individualized dynamics over time.

### 1.1 What this paper claims to contribute

Combining passive BCI, neurofeedback, adaptive human–machine systems, digital twins, and closed-loop control is not by itself a novel contribution. We therefore state the contribution narrowly. Section 4 gives the detailed comparison with existing architectures; the claim is that the contribution lies in five commitments, not in the components:

1. **A target-region objective rather than a classification objective**. Most cognitive-state systems are evaluated on how accurately they *label* the current state. We define the objective over a region of latent state space and evaluate four dynamic quantities—entry latency, dwell time, recovery latency, and drift magnitude—which are properties of the closed loop rather than of the classifier.
2. **Prediction of transitions, not detection of states, as the trigger**. The controller acts on the forecast *p*(*x*_*t*+Δ_∣*x*_*t*_, *u*_*t*_, τ_*t*_); the decisive quantity is drift *lead time*, making “how early” a first-class endpoint alongside “how accurate.”
3. **Burden as a constrained cost**. Intervention cost enters the objective explicitly, so a system restoring performance at unacceptable interruption or fatigue cost is scored a failure.
4. **The longitudinal individualized model as a separately falsifiable layer**. The twin carries its own null hypothesis (Section 9.7) and can fail while the rest of the system succeeds.
5. **An explicit separation of predictive from causal claims**, with stated evidentiary requirements for each (Section 12).

We claim no novelty for multimodal sensing, latent-state inference, state-conditioned intervention, or personalization taken individually; each has substantial prior art, cited throughout.

### 1.2 Terminology

The primary technical term used throughout is *closed-loop cognitive state control* (CLCSC). The term *supercognition* is retained only as an informal shorthand, and its scope is deliberately narrow: it denotes real-time control over access to the highest available cognitive state for a particular individual and task under safety and feasibility constraints. It does not denote omniscience, thought reading, unlimited memory, trait-level intelligence amplification, or transhuman enhancement. Section 2.3 gives the rationale for the change and the relationship between the two terms.

### 1.3 Structure

Section 2 states scope and terminology; Section 3 develops the background and makes the assumptions explicit; Section 4 differentiates the proposal from existing architectures; Sections 5–6 give the formal problem and objective, including how the objective would be estimated; Section 7 specifies candidate computational implementations; Section 8 presents the architecture; Section 9 specifies the personal neural twin; Section 10 discusses foundation and agentic AI; Section 11 engages the literature challenging the framework; Section 12 separates predictive from causal claims; Section 13 gives operational definitions; Section 14 assesses feasibility; Section 15 gives application scenarios; Section 16 specifies validation; Section 17 addresses ethics and governance; Sections 18–21 give discussion, limitations, roadmap, and conclusions.

## 2 Scope, article type, and terminology

### 2.1 Scope and article type

This is a hypothesis and conceptual framework paper introducing a testable theory and architecture, not a completed device, trial, or dataset. No human participants, animal subjects, empirical measurements, or simulations are reported; the earlier simulation-style validator has been removed because a toy validator with synthetic data creates a false impression of evidence.

The framework is within scope for neuroergonomics and bioengineering for three reasons, and addresses several of the open challenges identified for the field (Fairclough and Lotte, 2020). It concerns neurotechnology and human–machine systems; it defines a sensing–control architecture implementable with wearable or non-invasive bioelectronic systems; and it is translational in orientation, with the long-term goal of a platform for improving human performance reliability and the immediate goal of a falsifiable engineering model.

The central question is:

Can individualized closed-loop sensing and intervention increase reliable access to high-performing cognitive states, relative to unassisted performance and to open-loop assistance, at acceptable burden?

The answer remains the object of future validation.

### 2.2 What would make the framework false

Stating the falsifiers early is a scope commitment. If high-performing states show no recurring, individually stable signatures, state inference fails (Section 11.1). If departures are not detectable before performance loss with usable lead time, early correction fails. If state-conditioned interventions do not alter transition probabilities beyond sham, generic prompting, expectancy, and ordinary rest, closed-loop control fails (Section 12). If longitudinal individualized modeling does not outperform population and non-adaptive models after adequate data, the twin fails (Section 9.7). And if the burden of the loop exceeds its benefit, the system fails even when every component works.

### 2.3 Terminology: from “supercognition” to closed-loop cognitive state control

The label *supercognition* evokes intelligence amplification, transhuman enhancement, or extraordinary cognition—connotations far broader than the framework proposes and which it explicitly rejects. That is a real cost: a term requiring immediate qualification in every use is a poor technical term. We have therefore adopted *closed-loop cognitive state control* as the primary designation. It is descriptive, maps onto the control-theoretic formulation in Sections 5–6, and locates the work within neuroergonomics and neuroadaptive technology rather than enhancement discourse. *Supercognition* is retained as a shorthand and as a keyword for discoverability, but no argument here depends on it: where both appear, CLCSC is the definition and supercognition the label.

### 2.4 Relationship to adjacent constructs

The framework must also be distinguished from constructs it is easily confused with (Table 1). The differentiator is not the sensing modality or the intervention but the *objective*: CLCSC targets reliability of access to a state region over time—entry, dwell, recovery, drift—under an explicit burden constraint.

**Table 1:** Relationship of closed-loop cognitive state control to adjacent constructs.

Construct	Primary target	Time scale	Closed loop?	Individualized dynamics?	Key difference from CLCSC
Cognitive enhancement (trait)	Trait-level capacity: reasoning, working-memory capacity	Weeks to years	No	Rarely	Targets capacity, not access; CLCSC assumes capacity is fixed on the control time scale
Cognitive optimization/training	Task-specific skill and strategy	Weeks	No	Partly	Optimizes what the person can do, not whether the enabling state is currently occupied; far transfer is contested (Simons et al., 2016; Melby-Lervåg et al., 2016)
Cognitive augmentation	Offloading or extending cognition via external tools	Task duration	Sometimes	Rarely	Changes the task–agent division of labor; CLCSC changes the operator's state, leaving the task unchanged
Flow-state enhancement	Induction of a specific subjective experience	Minutes to hours	Rarely	Rarely	Targets one hypothesized state whose neural basis remains contested (Alameda et al., 2022; Gold and Ciorciari, 2020); CLCSC targets an empirically identified performance region that need not correspond to flow
Adaptive neurofeedback	Self-regulation of a designated neural feature	Sessions	Yes, within session	Partly	The controlled variable is a *signal feature* the user learns to regulate; in CLCSC the controlled variable is a *latent state*, and the system, not the user, selects the action
Passive BCI/neuroadaptive technology	System adaptation to an inferred user state	Seconds	Yes	Rarely	Closest prior art (Zander and Kothe, 2011; Zander et al., 2016; Krol et al., 2020); differs in that CLCSC forecasts transitions, optimizes dwell and recovery rather than momentary fit, and carries a longitudinal individual model
Adaptive automation	Allocation of function between human and machine	Seconds to minutes	Yes	Rarely	Modifies task load to fit the operator's state (Wilson and Russell, 2007; Parasuraman et al., 2000; Byrne and Parasuraman, 1996; Aricò et al., 2016); CLCSC may also act on the operator's state directly, and treats the state trajectory as the controlled output
Peak performance regulation (applied sport/performance psychology)	Pre-performance routines, arousal regulation	Minutes to hours	Open loop, self-administered	By coaching	Relies on self-report and coach judgment rather than real-time inference; CLCSC formalizes the same intuition as a measurable control problem

## 3 Conceptual background

### 3.1 Trait capacity vs. state access

Trait-level measures aggregate developmental history, education, reasoning ability, and working-memory capacity. They matter, but are not real-time control variables: a closed-loop device cannot redesign a user's developmental history within a work session. State variables are different. Arousal, attention, fatigue, cognitive noise, task engagement, stress load, and recovery trajectory vary over seconds to minutes and are partially observable, potentially predictable, and potentially controllable. A user may have sufficient ability to solve a problem yet not be in the state in which that ability is expressed, and a system that improves entry into that state, extends dwell, or shortens recovery may improve output without changing trait intelligence.

Open-loop intervention is limited because the same stimulus can help in one state and harm in another: a reminder may restore focus during drift but fragment stable engagement; a breathing protocol may reduce stress during overload but add friction when under-engaged. State awareness is therefore the precondition for the right intervention at the right time—the argument that also motivates just-in-time adaptive interventions, whose decision rules are defined over a tailoring variable representing momentary state and receptivity (Nahum-Shani et al., 2018).

### 3.2 What kind of thing is a cognitive state?

The framework depends on the assumption that high-performance cognitive states are identifiable latent entities that can be inferred and, to some degree, controlled. Four questions must be answered explicitly, because different answers imply different estimators, validation designs, and failure modes.

#### 3.2.1 Discrete or continuous?

Categorical accounts treat cognition as occupying qualitatively distinct regimes; dimensional accounts treat it as a point in a low-dimensional space, commonly spanned by arousal and task engagement (Dehais et al., 2020; Aston-Jones and Cohen, 2005). Evidence favors neither decisively: sustained-attention research describes quasi-discrete alternation between “in the zone” and “out of the zone” periods while also modeling attention as continuously fluctuating (Esterman and Rothlein, 2019), and mind-wandering is characterized as a graded dynamic process rather than a binary event (Christoff et al., 2016).

#### 3.2.2 Stable or context-dependent?

Physiological signatures of workload and fatigue are reliably modulated within a session but shift across sessions, days, devices, and environments (Wu et al., 2022; Borghini et al., 2014). Non-stationarity is the normal case, not an artifact.

#### 3.2.3 Task-general or task-specific?

Task-independent workload classifiers have been attempted, but cross-task transfer remains the weakest link in cognitive-state decoding (Wu et al., 2022), and multiple-resource accounts imply that “load” is not a single scalar (Wickens, 2008).

#### 3.2.4 Individual-specific or population-general?

Individual differences in cognitive tasks are unreliable even for well-established paradigms (Hedge et al., 2018), and brain–behavior associations from small samples are unstable (Marek et al., 2022). This argues *for* individualized modeling—population effects may describe no particular person—while warning that individualized estimates need far more within-person data than is usually collected.

### 3.3 Assumptions adopted by this framework

Rather than resolve these debates, we state the assumptions the framework adopts, so each can be tested and fail independently.

- **A1 (Weak discreteness)**. The latent space *X*_*i*_ is continuous, but performance-relevant structure is well approximated by a small number of recurrent regimes with individual-specific occupancy. The framework therefore uses switching or semi-Markov models over a continuous latent space rather than a hard classifier. *Fails if* regime-switching models do not outperform continuous-only models on held-out prediction of performance.
- **A2 (Within-person recurrence)**. For a fixed individual and task class, high-performing intervals recur with sufficiently similar multimodal signatures to be identified across sessions. *Fails if* within-person, cross-session identification does not exceed chance after adequate data (Section 11.1).
- **A3 (Task conditioning)**. State structure is defined *within* a task class, not globally. The framework does not assume a task-general state ontology and does not require cross-task transfer to succeed. *Fails* only if within-task-class structure is itself unstable.
- **A4 (Partial observability with informative precursors)**. Departures from a target region are preceded by measurable changes with non-zero lead time. *Fails if* the distribution of lead times is concentrated at or below the actuation latency of any admissible intervention.
- **A5 (Bounded controllability)**. Interventions shift transition probabilities rather than setting the state. The framework claims stochastic influence, not deterministic control. *Fails if* state-conditioned intervention effects are indistinguishable from state-independent effects.

A1–A5 are deliberately weak: in particular, the framework requires no fixed, shared, or task-general state ontology.

### 3.4 Grounding the provisional state labels

Ten candidate states—baseline engagement, under-arousal, deep focus, flow formation, overload, fatigue onset, affective interference, distraction cascade, unstable recovery, pressure-induced fragmentation—map onto established constructs in psychology and neuroscience, and Table 2 anchors each to them. Two caveats apply. The labels remain *provisional scaffolding*: under A1–A3 a mature system learns individualized structure rather than imposing this list, and they are useful only insofar as they predict performance and intervention response. And the listed correlates are candidate indicators from group-level literature, whose within-person reliability is what Section 11.1 identifies as unresolved.

**Table 2:** Provisional state labels, the constructs they correspond to, candidate correlates, and the principal interpretive caution for each.

Provisional label	Corresponding established construct	Candidate correlates reported in the literature	Principal caution
Baseline engagement	Task engagement in the arousal × engagement space (Dehais et al., 2020)	Stable task-evoked activity; moderate autonomic arousal	Baseline is person- and task-relative, not absolute
Under-arousal	Low arousal limb of the arousal–performance relation (Yerkes and Dodson, 1908; Aston-Jones and Cohen, 2005); effort withdrawal (Dehais et al., 2020)	Elevated posterior alpha, reduced pupil-linked arousal, increased response variability (Pfurtscheller and Lopes da Silva, 1999; Aston-Jones and Cohen, 2005; Mathôt, 2018)	The inverted-U is a descriptive generalization, not a mechanism
Deep focus	Sustained focused attention; “in the zone” periods in sustained-attention models (Esterman and Rothlein, 2019); cognitive control engagement (Shenhav et al., 2013)	Reduced response variability, task-positive network engagement, reduced default-mode engagement (Christoff et al., 2016)	Concentration and neural synchrony are not interchangeable; dopaminergic accounts are inferential in humans
Flow formation/flow-like engagement	Flow (Csikszentmihalyi, 1990); challenge–skill balance	Heterogeneous and inconsistent across studies; frontal alpha and midline theta findings do not converge (Alameda et al., 2022; Peifer et al., 2022; Gold and Ciorciari, 2020)	Evidence for a distinct neural signature of flow is sparse and inconclusive (Alameda et al., 2022); treat as hypothesis, not as a target
Overload	Mental workload exceeding available resources (Wickens, 2008); workload in the neuroergonomic space (Dehais et al., 2020; Diarra et al., 2025)	Prefrontal oxygenation change, frontal midline theta increase, pupil dilation, HRV suppression (Dehais et al., 2020; Diarra et al., 2025; Borghini et al., 2014)	Workload lacks a consensual operational definition (Diarra et al., 2025); overlaps strongly with stress (Diarra et al., 2025)
Fatigue onset	Vigilance decrement and time-on-task fatigue (Warm et al., 2008; Lim and Dinges, 2008)	Alpha and theta power increase, blink dynamics, slowing and increased variability (Lim and Dinges, 2008; Borghini et al., 2014)	Vigilance decrement reflects resource depletion *and* stress (Warm et al., 2008); not reducible to sleepiness
Affective interference	Emotional interference with cognitive control	Autonomic reactivity, asymmetry measures	Weakest construct in the list; affect decoding from biometrics is contested and regulated (Section 17.7)
Distraction cascade	Attentional capture and switching residue	Increased switch cost, gaze dispersion, rework	Behavioral rather than neural in origin; may be measurable from task telemetry alone
Unstable recovery	Post-interruption resumption cost	Prolonged resumption lag, elevated variability after return	Confounded with task difficulty at the resumption point
Pressure-induced fragmentation	Performance under pressure; choking; anxiety-driven control disruption	Elevated sympathetic markers with degraded performance despite maintained effort	Requires an independent manipulation of pressure to be identified at all

### 3.5 Closed-loop neuroengineering: the relevant precedents

Closed-loop neuroengineering follows one pattern: sense a biological state, infer a latent condition, select an intervention, observe the response, update the model. The strongest existing demonstrations are clinical, spanning stimulation, rehabilitation, and adaptive prosthetics (Young et al., 2014; Shenoy and Carmena, 2014). Responsive neurostimulation for epilepsy (Morrell, 2011) and adaptive deep brain stimulation (DBS) for Parkinson's disease (Oehrn et al., 2024; Little et al., 2013) show that closed-loop control on a physiological biomarker can outperform open-loop stimulation in a blinded design; the 2024 randomized crossover trial of chronic adaptive DBS is the clearest existing proof that the *closed-loop* element, and not merely the intervention, carries benefit (Oehrn et al., 2024).

In human–machine systems, adaptive automation triggered by physiological indices has improved performance in uninhabited-vehicle supervision (Wilson and Russell, 2007) and in realistic air traffic control (Aricò et al., 2016), and neuroadaptive technology has demonstrated implicit control derived from event-related brain responses without volitional user input (Zander et al., 2016; Krol et al., 2020). These are the closest existing analogs to the present proposal, and Section 4 compares against them directly.

### 3.6 From cognitive enhancement to state sovereignty

A more precise phrase for the objective is *cognitive state sovereignty*: measurable, individualized control over access to one's own high-functioning cognitive regimes. The goal is not to impose an externally defined ideal state. It is to help a user recognize, enter, maintain, and recover the states in which that user's own performance is best, while preserving consent, reversibility, privacy, and biological limits. This is a normative commitment with architectural consequences, developed in Section 17.

## 4 Relation to existing closed-loop architectures

This section establishes what, if anything, is new, by comparison against the closest existing architectures.

### 4.1 Comparison

Table 3 compares the proposed framework with six families of existing systems across the dimensions that matter for a control formulation.

**Table 3:** Existing cognitive-state and closed-loop architectures compared with the proposed framework.

Dimension	Passive BCI/neuroadaptive technology (Zander and Kothe, 2011; Zander et al., 2016; Krol et al., 2020)	Adaptive automation (Wilson and Russell, 2007; Parasuraman et al., 2000; Byrne and Parasuraman, 1996; Aricò et al., 2016)	Neurofeedback (Sitaram et al., 2017; Ros et al., 2020; Thibault et al., 2016)	Adaptive DBS/RNS (Oehrn et al., 2024; Little et al., 2013; Morrell, 2011)	JITAI in mobile health (Nahum-Shani et al., 2018; Klasnja et al., 2015)	Human digital twins (National Academies of Sciences, Engineering, and Medicine, 2024; Katsoulakis et al., 2024; Laubenbacher et al., 2024)	Proposed framework (CLCSC)
Sensing modality	EEG, often single-modality	EEG, cardiac, ocular	EEG or fMRI, single designated feature	Intracranial LFP/ECoG	Phone, wearable, self-report	Clinical, imaging, omics; rarely continuous neural	Multimodal: neural, ocular, cardiac, motor, task telemetry, context
Controlled variable	System interface or task allocation	Function allocation	A signal feature the user self-regulates	Stimulation amplitude	Delivery of a behavioral prompt	Usually none: model is descriptive	Latent state trajectory of the operator
Prediction of transitions	Rare	Rare	No	Emerging	Occasional receptivity models	Sometimes, for disease course	Central; drift lead time is a primary endpoint
Personalization	Per-session calibration	Threshold tuning	Per-user protocol selection	Per-patient biomarker identification (Oehrn et al., 2024)	Decision rules with tailoring variables	Definitional requirement (National Academies of Sciences, Engineering, and Medicine, 2024)	Hierarchical: population prior to individual posterior, updated longitudinally
Longitudinal learning across days	Rare	Rare	Across training sessions, for the user not the model	Increasingly, at-home chronic recordings (Oehrn et al., 2024)	Yes, via micro-randomization (Klasnja et al., 2015)	Aspirational; rarely realized (Katsoulakis et al., 2024)	First-class layer with its own falsifier (Section 9.7)
Objective	Momentary fit of system to state	Maintain workload in a band	Change a signal feature	Suppress a pathological biomarker	Proximal behavioral outcome	Predict clinical course	Entry, dwell, recovery, drift, under a burden constraint
Burden treated as a cost	Implicit	Implicit	Not applicable	Battery and side effects	Notification fatigue, discussed	Not applicable	Explicit term in the objective and a hard ceiling
Causal claim status	Mixed; often correlational	Some randomized evidence (Wilson and Russell, 2007; Aricò et al., 2016)	Contested; sham control central (Ros et al., 2020; Thibault et al., 2016)	Randomized, blinded evidence exists (Oehrn et al., 2024)	Micro-randomized, causal by design (Klasnja et al., 2015; Liao et al., 2016)	Usually none	Separated explicitly by claim type (Section 12)

“Prediction” means forecasting a future state or transition, not classifying the present one.

### 4.2 What is new, and what is not

#### 4.2.1 Not new

Multimodal physiological sensing of cognitive state; latent-state inference from physiological data; state-conditioned adaptation of an interface or task; per-user calibration; the digital-twin metaphor. Each has substantial prior art cited above, and the framework depends on that prior art rather than replacing it.

#### 4.2.2 New, as a combination and as a commitment

First, the reformulation of the goal as occupancy of a *target region* of latent state space, with entry, dwell, recovery, and drift as the primary endpoints, rather than as classification accuracy or momentary interface fit. Second, the elevation of *transition forecasting with quantified lead time* to the control trigger, which changes what the inference layer must deliver and what the validation must measure. Third, the explicit inclusion of intervention burden in the objective and as a hard constraint, so that the trade-off adaptive systems usually leave implicit becomes an evaluated quantity. Fourth, the treatment of the longitudinal individual model as an independently falsifiable component with its own null hypothesis and its own failure modes (Section 9). Fifth, an explicit map from each claim to its evidentiary requirement, separating association, prediction, and causal control (Section 12).

#### 4.2.3 Honest assessment

Points one to three reformulate an objective existing systems address implicitly; their value is that they make it measurable and comparable across systems, not that the underlying idea is unprecedented. Point four is the element most likely to be a genuine theoretical contribution and also the one with the least supporting evidence, which is why Section 9 specifies it and Section 9.7 states how it dies. Point five is methodological hygiene rather than novelty, though Section 11 suggests it is not yet standard practice here.

## 5 Formal problem statement

### 5.1 Notation

Let an individual *i* occupy a latent cognitive state *x*_*t*_ ∈ *X*_*i*_ at time *t*, where *X*_*i*_ is the feasible state space for that person. Let *y*_*t*_ ∈ *Y* denote observable data streams, including neural measurements, eye dynamics, heart-rate variability, respiration, motor timing, speech or typing rhythm, task telemetry, context, sleep history, and self-report where appropriate. Let τ_*t*_ ∈ *T* denote the task context, and *u*_*t*_ ∈ *S*_*i*_ an intervention drawn from the individual's admissible set.

Performance is represented by a vector-valued functional


$$\begin{array}{l} P_{i}\left(x_{t},\tau_{t}\right)\in {\mathbb{R}}^{m}, \end{array}$$

whose components may include accuracy, response latency, response variability, error rate, working-memory stability, attentional persistence, recovery time, and pressure resilience.

This is a partially observable stochastic control problem: given *y*_1:*t*_ and τ_1:*t*_, infer *x*_*t*_, forecast transitions, and select *u*_*t*_ to improve occupancy of high-performing states under safety and burden constraints. Formally it is a partially observable Markov decision process (POMDP) 〈*X*_*i*_, *S*_*i*_, *T*_*i*_, *R*_*i*_, *Y, O*_*i*_, γ〉 whose transition kernel *T*_*i*_ and observation model *O*_*i*_ are individual-specific and initially unknown—and are what the personal neural twin estimates.

#### 5.1.1 Definition 5.1 (Closed-loop cognitive state control; informally, supercognition)

*Closed-loop cognitive state control is the capability of a sensing-and-intervention system to increase, relative to matched unassisted and open-loop conditions, the reliability with which an individual enters, sustains, and recovers that individual's highest available task-specific cognitive state, where reliability is measured jointly by entry latency, dwell time, recovery latency, and drift magnitude, and where the improvement is achieved within stated safety, consent, and burden constraints*.

Three bounds are built in: the target is individualized rather than universal, task-specific rather than globally optimal, and constrained by feasible biology, consent, safety, and burden. The definition is comparative—improvement is defined against specified control conditions—so it is measurable rather than aspirational.

## 6 Target state and optimization objective

### 6.1 Objective function and target region

For individual *i* and task class τ, let *F*_*i*, τ_ ⊆ *X*_*i*_ be the safe feasible state set. Let *J*_*i*_(*x*, τ) be a scalar objective derived from performance- and safety-relevant variables:


$$\begin{array}{r} J_{i}\left(x,\tau \right)=w_{1}A\left(x,\tau \right)-w_{2}E\left(x,\tau \right)-w_{3}L\left(x,\tau \right)+w_{4}S\left(x,\tau \right)+\\ w_{5}R\left(x,\tau \right)-w_{6}B\left(x,\tau \;\right), \end{array}$$

where *A* is accuracy or task success, *E* is error rate, *L* is latency, *S* is stability, *R* is recovery efficiency, *B* is burden or adverse-state cost, and *w*_*k*_ ≥ 0 are task-dependent weights.

#### 6.1.1 Definition 6.1 (Highest available task-specific cognitive state)

The target for individual *i* and task class τ is


$$\begin{array}{l} x_{i,\tau }^{⋆}=\text{arg}\underset{x\in F_{i,\tau }}{\max}J_{i}\left(x,\tau \right), \end{array}$$

or, more realistically, a target region $N\left(x_{i,\tau }^{⋆}\right)=\left\{x\in F_{i,\tau }:J_{i}\left(x,\tau \right)\geq J_{i}\left(x_{i,\tau }^{⋆}\right)-\epsilon \right\}$ containing states that satisfy a performance threshold and safety constraint. The region formulation is preferable to a point estimate because cognition is noisy, measurement is imperfect, and high performance may correspond to a family of nearby latent configurations. The tolerance ϵ is a design parameter with an operational meaning: it is the performance decrement the deployment is willing to ignore, and it should be set to the minimum operationally meaningful difference specified in advance (Section 16.4).

Relating this to established constructs: $x_{i,\tau }^{⋆}$ is *not* flow, whose neural basis remains contested (Alameda et al., 2022); nor minimal workload, since the workload minimizing effort is frequently not the workload maximizing performance (Dehais et al., 2020; Wickens, 2008); nor maximal attentional engagement, since over-engagement is associated with perseveration and reduced flexibility (Dehais et al., 2020). It is defined operationally by *J*_*i*_; its relation to flow, cognitive control, attentional engagement, mental workload, and expertise is an empirical question the validation program is designed to answer rather than assume.

### 6.2 Outcome variables

Four dynamic quantities define the objective:

- *T*_*entry*_: time required to enter $N\left(x_{i,\tau }^{⋆}\right)$ from task onset or from a defined perturbation;
- *T*_*dwell*_: cumulative time within $N\left(x_{i,\tau }^{⋆}\;\right)$;
- *T*_*recovery*_: time to return to $N\left(x_{i,\tau }^{⋆}\right)$ after departure;
- *D*_*drift*_: magnitude and rate of departure from the target region.

A closed-loop policy π maps belief state and context to interventions. The objective is


$$\begin{array}{r} \underset{\pi }{\max}\;{𝔼}_{\pi }\\ \left[\alpha T_{\text{dwell}}-\beta T_{\text{entry}}-\gamma T_{\text{recovery}}-\delta D_{\text{drift}}-\lambda \underset{t}{\sum }C\left(u_{t}\right)\right], \end{array}$$

subject to *u*_*t*_ ∈ *S*_*i*_, *x*_*t*_ ∈ *F*_*i*, τ_, and user-defined safety and consent constraints, where *C*(*u*_*t*_) is intervention burden.

### 6.3 How this objective would actually be estimated

The formulation above is symbolic until each unobservable quantity is tied to a procedure. This section specifies how each would be obtained, which is the difference between a scaffold and a specification.

#### 6.3.1 The latent argument

*J*_*i*_ depends on a latent state and cannot be evaluated directly. The system therefore optimizes the expected objective under the posterior, *E*_*x* ~ *p*(_*x*_*t*__ ∣ *y*_1:*t*_, τ_1:*t*_)_[*J*_*i*_(*x*, τ)], computable from the inference layer. Region membership becomes a probability, $\mathrm{Pr}\left(x_{t}\in N\left(x_{i,\tau }^{⋆}\right)|y_{1:t}\right)$, and the four outcome variables are computed from the posterior occupancy trace using a stated threshold and a stated minimum dwell duration, so that single-sample excursions are not counted.

#### 6.3.2 Defining the region empirically

$N\left(x_{i,\tau }^{⋆}\right)$ is identified, not assumed. During characterization, concurrent performance *P*_*i*_ is recorded alongside *y*_*t*_, and the region is estimated as the set of latent configurations whose posterior predictive performance exceeds a preregistered threshold—a level set of a fitted state-to-performance mapping. The target region is thus a derived, individually estimated quantity with confidence bounds, not a label assigned by an experimenter.

#### 6.3.3 Weights

The weights *w*_*k*_ and α, β, γ, δ, λ encode a value judgment about trade-offs and should not be set silently by the engineer. Three defensible options exist: (i) *task-derived*, by fitting weights so that *J*_*i*_ best predicts an externally defined operational outcome, such as detected errors in a simulator; (ii) *preference-elicited*, using discrete-choice or conjoint methods in which the user chooses between described state–burden profiles; or (iii) *inverse-reinforcement-learning*, inferring weights from the user's own accept/dismiss behavior toward interventions. Option (iii) is attractive but self-confirming and must be regularized against the degenerate solution of never intervening. We recommend (i) for validation studies, where the operational outcome is defined by the study, and (ii) for deployment, where the user's own trade-off should govern.

#### 6.3.4 Burden

*C*(*u*_*t*_) is measurable rather than notional: dismissal rate, post-intervention resumption lag, subjective intrusiveness ratings sampled by ecological momentary assessment, and the cumulative time cost of the intervention itself. A burden ceiling should be preregistered as a constraint, not traded off freely against performance.

#### 6.3.5 Policy evaluation without deployment

Because deploying an untested policy on a human operator carries risk, candidate policies should first be evaluated offline on logged data using off-policy evaluation with importance weighting or doubly robust estimators, which requires that the data-collection policy be stochastic and its randomization probabilities logged (Levine et al., 2020). This is one of several reasons the validation design in Section 16 randomizes intervention delivery at the decision point.

#### 6.3.6 Causal effect of an intervention

The quantity the controller actually needs is not a correlation between state and outcome but the *causal excursion effect* of delivering *u*_*t*_ vs. withholding it in a given state. This is precisely what a micro-randomized trial estimates: intervention options are randomized at each of many decision points within each person, and time-varying moderated effects are estimated from the resulting data (Klasnja et al., 2015; Liao et al., 2016). We adopt this as the framework's primary causal estimation strategy (Sections 12 and 16).

## 7 Candidate computational implementations

This section specifies how latent states would be estimated, how transition probabilities would be modeled, and which methods are appropriate. The intent is not to prescribe one implementation but to identify the candidate families, their data and latency requirements, and their failure modes, so that two independent groups could build comparable systems.

### 7.1 Layer 2: latent-state inference

The inference layer estimates the posterior *p*(*x*_*t*_ ∣ *y*_1:*t*_, τ_1:*t*_). Four families are appropriate, and they are not mutually exclusive.

#### 7.1.1 Hidden Markov and hidden semi-Markov models

Hidden Markov and hidden semi-Markov models are the natural starting point under A1; the semi-Markov variant parameterizes each regime's duration distribution directly, which matters because dwell time is an endpoint rather than a nuisance. Its limitations are the geometric dwell-time assumption in the plain hidden Markov model (HMM) case and the need to fix the state count, which nonparametric priors relax.

#### 7.1.2 Linear and switching state-space models with Bayesian filtering

Where the latent state is better treated as continuous, a linear-Gaussian state-space model with a Kalman filter gives a real-time posterior at negligible cost and has an established record in neural decoding (Wu et al., 2006; Gilja et al., 2012). Recurrent switching linear dynamical systems represent the state as continuous while allowing discrete regime changes with state-dependent switching probabilities (Linderman et al., 2017), the closest formal match to A1. Non-linear or non-Gaussian variants require unscented Kalman or particle filtering at higher cost.

#### 7.1.3 Discriminative and representation-learning approaches

Where labels exist, regularized linear models on covariance features remain strong baselines, and Riemannian methods are the current standard because they respect the geometry of the feature space and transfer comparatively well across sessions (Congedo et al., 2017). Deep learning's median gain over traditional pipelines is real but modest, and its reproducibility problems are a design constraint rather than a footnote (Roy et al., 2019). Self-supervised pretraining on unlabeled recordings matters because cognitive-state labels are scarce and expensive (Banville et al., 2021).

#### 7.1.4 Hybrid architecture, recommended

We recommend a learned encoder mapping raw multimodal windows to a low-dimensional feature vector, with a switching state-space model operating on that vector. This separates representation learning, which benefits from large unlabeled corpora, from dynamics modeling, where individual-specific structure lives and where interpretability and uncertainty quantification are needed.

#### 7.1.5 The label problem

Supervised state inference presupposes labels that do not exist in nature. Four imperfect sources are available: experimentally induced conditions; concurrent performance as a proxy, which conflates state with ability; probe-based self-report, which is intrusive and reactive; and cognitive or affective probing, in which the system inserts brief context-sensitive probes and reads the evoked response (Krol et al., 2020). Performance-anchored definition (Section 6.3) plus sparse probing is the most defensible combination, and the choice of label source is a substantive scientific commitment rather than a preprocessing detail.

### 7.2 Layer 3: transition prediction

The controller requires *p*(*x*_*t*+Δ_ ∣ *x*_*t*_, *u*_*t*_, τ_*t*_) and, more specifically, the distribution of *time until departure* from the target region. Three formulations are appropriate.

#### 7.2.1 Survival and hazard models

Survival and hazard models treat departure from $N\left(x_{i,\tau }^{⋆}\right)$ as an event whose hazard depends on the belief state, time on task, and context. This beats naive classification because it handles censoring, models time-to-event directly, and yields the lead-time quantity the objective needs; discrete-time hazard models with person-level random effects are a practical default.

#### 7.2.2 Sequence models

Recurrent networks and temporal transformers forecast multivariate physiological trajectories over seconds to minutes and can condition on planned interventions, and they are the natural way to exploit pretrained representations (Section 10). Their weakness is calibration: they are frequently overconfident, and since the controller consumes probabilities rather than point predictions, *post-hoc* calibration is mandatory (Guo et al., 2017).

#### 7.2.3 Gaussian-process and state-space forecasts

Gaussian-process and state-space forecasts suit the low-data regime of early deployment, quantifying uncertainty natively and degrading gracefully toward the prior.

Closed-loop EEG decoding with deep models has been assembled end-to-end, with real-time classification of attention and memory states driving neurofeedback (Khan and Rehman, 2025). Such reports show the pipeline can run within latency; they do not establish durable transfer, being typically single-session, single-site, and evaluated without the open-loop control arm Section 16.1 requires. That gap is the translational question this framework makes testable.

#### 7.2.4 Lead time is the endpoint

The operational metric is not forecast accuracy in the abstract but the achieved distribution of lead time at a fixed false-alarm rate, evaluated against intervention actuation latency (Section 14.3). An accurate forecast arriving 400 ms before the event is useless if the shortest admissible intervention needs 2 s to take effect.

### 7.3 Layer 4: policy

#### 7.3.1 Contextual bandits

*Contextual bandits* suit short-horizon effects where the question is which action to take in which state. Thompson sampling with a linear or Gaussian-process reward model over the belief state is a practical default and generates the randomization needed for causal estimation and off-policy evaluation.

#### 7.3.2 Model-predictive control

Model-predictive control becomes appropriate once a usable transition model exists, since it optimizes over a horizon, respects explicit constraints on *F*_*i*, τ_ and burden, and is re-solved at each decision point.

#### 7.3.3 Offline and batch-constrained reinforcement learning

Offline and batch-constrained reinforcement learning suits accumulated longitudinal logs but risks extrapolation error when the learned policy departs from the data-collection distribution (Levine et al., 2020). Policy-optimization work in multi-agent reinforcement learning, including hybrid value-selection and self-attention architectures, offers action-selection methods relevant to the team-level extensions in Section 10.3 (Rehman et al., 2021; Younas et al., 2025); it was developed in simulated games where sample cost is negligible and the actuator is not a person, so its sample-efficiency results should not be read as achievable here. Because the actuator is a human being, policy improvement should stay near the logged policy, and any learned policy should pass off-policy evaluation against a preregistered threshold before deployment.

#### 7.3.4 Safety layer

Regardless of policy family, a non-learned safety layer sits between policy output and delivery, enforcing *u*_*t*_ ∈ *S*_*i*_, the burden ceiling, and refractory periods, and abstaining when the state posterior is too diffuse to support a decision. Abstention under uncertainty is a design requirement, not a degraded mode.

### 7.4 Layer 5: personalization machinery

#### 7.4.1 Hierarchical Bayesian partial pooling

Hierarchical Bayesian partial pooling is the principled solution to the cold-start problem: individual parameters are drawn from a population distribution, so a new user begins at the population mean and moves toward their own estimate at a rate governed by their data (Gelman and Hill, 2007). This addresses the tension in Section 3.2: individual estimates are unreliable with little data, yet population estimates may describe no one.

#### 7.4.2 Meta-learning and transfer

Pretraining across users then adapting per user is the machine-learning analog. Euclidean or Riemannian alignment of covariance features before transfer is a cheap, well-established way to reduce inter-subject and inter-session shift (Wu et al., 2022; Congedo et al., 2017).

#### 7.4.3 Federated learning

Federated learning allows population-level improvement without centralizing raw neural data—a privacy requirement rather than a convenience in this domain (Rieke et al., 2020) (Section 17.2). It is not free: federated training carries communication and energy costs borne by battery-constrained wearables, where efficiency and scalability remain open (Shahid et al., 2026). It also reduces centralized exposure without eliminating inference risk from model updates, so it belongs alongside on-device processing and differential privacy, not in place of them.

### 7.5 Selection guidance

See Table 4 for Selection guidance.

**Table 4:** Candidate methods by layer, with data requirements, latency, and characteristic failure modes.

Layer	Method family	What it estimates	Within-person data need	Inference latency	Interpretability	Characteristic failure mode
2	HMM/HSMM	Discrete regime posterior with dwell distribution	Low–moderate	Milliseconds	High	State count mis-specification; label switching across sessions
2	Linear SSM + Kalman filter	Continuous latent trajectory	Low	Milliseconds	High	Model mismatch under non-linear dynamics
2	Switching linear dynamical system (Linderman et al., 2017)	Continuous state with discrete regimes	Moderate	Milliseconds–seconds	Moderate	Identifiability; sensitivity to initialization
2	Particle filter	Non-Gaussian posterior	Moderate	Seconds	Moderate	Particle degeneracy; compute cost on device
2	Riemannian covariance methods (Congedo et al., 2017)	Discriminative state estimate	Low–moderate	Milliseconds	Moderate	Degrades with heavy artifact contamination
2	Deep encoders/self-supervised (Roy et al., 2019; Banville et al., 2021)	Learned representation	High (or transfer)	Milliseconds–seconds	Low	Reproducibility and overfitting; poor cross-session transfer (Roy et al., 2019; Wu et al., 2022)
3	Discrete-time hazard model	Time to departure from target region	Moderate	Milliseconds	High	Proportionality assumption violated by context shifts
3	Recurrent/transformer forecaster	Multivariate trajectory	High	Milliseconds–seconds	Low	Overconfidence; requires explicit calibration (Guo et al., 2017)
3	Gaussian process	Short-horizon forecast with uncertainty	Low	Seconds	Moderate	Scaling with data volume
4	Contextual bandit (Thompson sampling)	Action value by state	Low, accumulating	Milliseconds	Moderate	Myopia; ignores delayed effects
4	Model-predictive control	Constrained action sequence	Requires transition model	Milliseconds–seconds	High	Inherits transition-model error
4	Offline/batch-constrained RL (Levine et al., 2020)	Long-horizon policy	High	Milliseconds	Low	Extrapolation error; unsafe off-support actions
5	Hierarchical Bayes (Gelman and Hill, 2007)	Individual parameters with shrinkage	Low, improving	Offline update	High	Over-shrinkage masks genuine individuality
5	Meta-learning/domain adaptation (Wu et al., 2022; Congedo et al., 2017)	Fast per-user adaptation	Low	Offline update	Low	Negative transfer from dissimilar source users
5	Federated learning (Rieke et al., 2020)	Population model without raw data sharing	Population-scale	Offline update	Low	Client drift; still leaks information without additional protection

“Within-person data” refers to labeled or performance-anchored data from the individual.

### 7.6 Identifiability

Two identifiability problems limit any implementation. First, such models are identifiable only up to relabeling and, for continuous latents, affine transformation; “the same state” across sessions therefore requires anchoring—most practically to performance or an induced condition—rather than following from the fit. Second, in a closed loop the intervention influences the data from which the model is subsequently estimated, so the system can converge to a self-confirming model that appears accurate while being wrong about counterfactuals. This is the cognitive-domain analog of the co-adaptation problem in neural prosthetics, where decoder and user adapt to one another simultaneously (Shenoy and Carmena, 2014). Persistent randomization at the decision point is the standard remedy and is another reason to adopt a micro-randomized design (Klasnja et al., 2015; Liao et al., 2016).

## 8 Proposed system architecture

The platform has five layers: multimodal sensing, latent-state inference, predictive transition modeling, closed-loop intervention, and a personal neural twin. Figure 1 shows the layers, the information flow between them, the outcome-feedback path that supplies the learning signal, and the constraint layer that bounds admissible actions.

**Figure 1:**
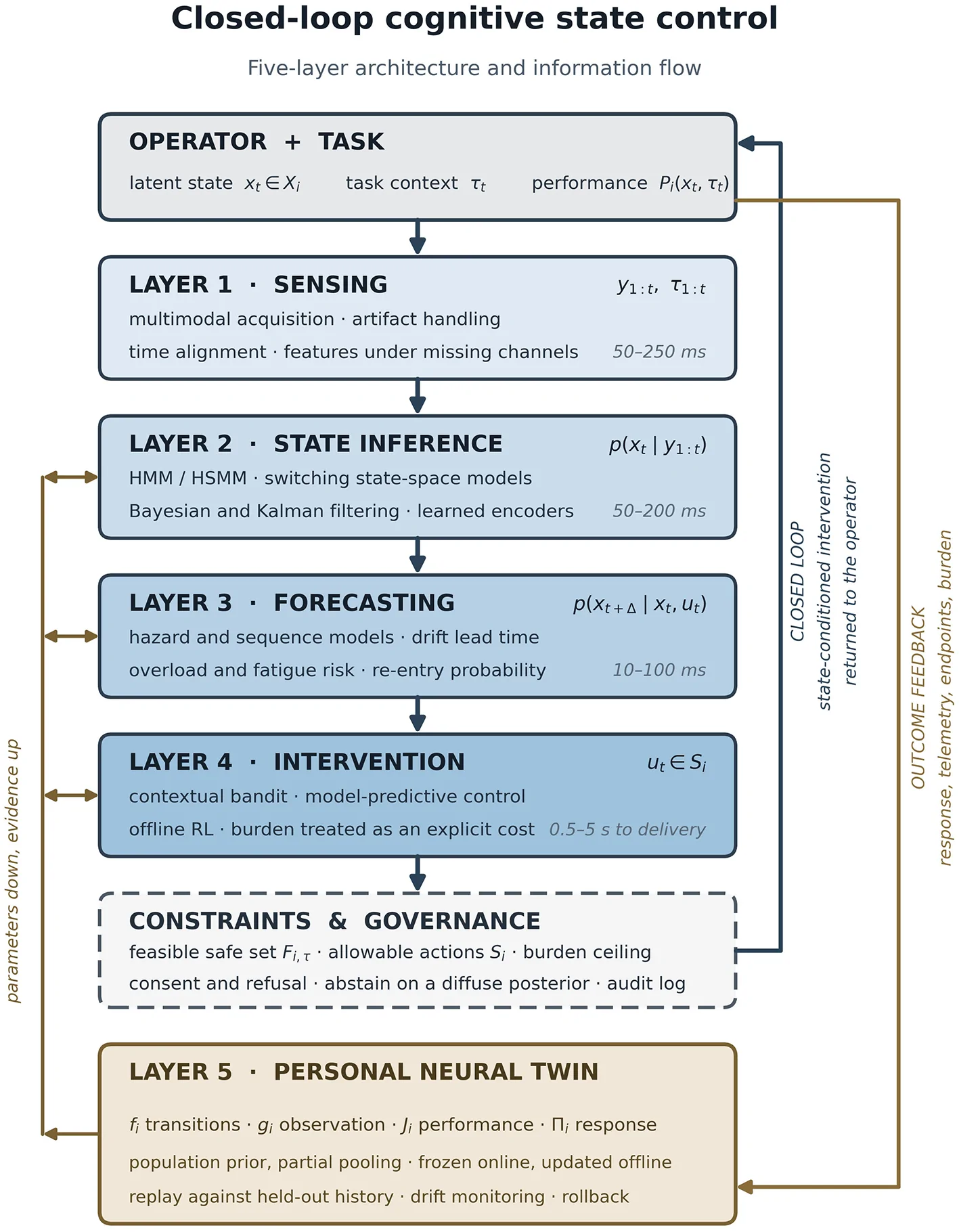
Closed-loop cognitive state control: five-layer architecture and information flow. The forward path carries signals from the operator through sensing, latent-state inference, transition forecasting, and intervention selection, and returns a state-conditioned intervention to the operator. The outcome-feedback path returns the observed response, task telemetry, and the four endpoints to the personal neural twin *M*_*i*_ = {*f*_*i*_, *g*_*i*_, *J*_*i*_, Π_*i*_}, which exchanges parameters and evidence with layers 2–4. The constraint layer bounds the admissible action set at every decision point and enforces abstention when the state posterior is too diffuse to support a decision. Indicative latencies are given per layer; the end-to-end budget is discussed in Section 14.3.

### 8.1 Layer 1: multimodal sensing

Candidate streams include electroencephalography, functional near-infrared spectroscopy, eye tracking and pupillometry, heart-rate variability, respiration, electrodermal activity, typing cadence, speech dynamics, task telemetry, movement microstructure, context, and brief self-report (Paradiso et al., 2005; Fairclough, 2009; Ayaz et al., 2011; Mullen et al., 2015; Mathôt, 2018; Shaffer and Ginsberg, 2017).

The principle is to maximize state information per unit burden, not sensor count: an accurate but intrusive sensor may degrade the very state it is meant to support, while a slightly less informative but persistently available stream may be more valuable for longitudinal control. Task telemetry deserves emphasis—it is free, unobtrusive, individually calibrated by construction, and for several states in Table 2 may carry more usable information than any physiological channel. The layer must handle asynchronous sampling, channel dropout, and artifact, modeling missingness rather than imputing it silently, since missingness is informative: motion artifact correlates with the state changes of interest.

### 8.2 Layer 2: latent-state inference

The inference layer estimates *p*(*x*_*t*_ ∣ *y*_1:*t*_, τ_1:*t*_) by the methods in Section 7.1. Its output is a posterior, not a label, and three properties are required: calibrated uncertainty, since the safety layer abstains on diffuse posteriors; stability under channel dropout, since an estimate that changes when a sensor detaches is not a state estimate; and per-session anchoring, since latent states are identifiable only up to relabeling (Section 7.6).

### 8.3 Layer 3: predictive transition modeling

Classification is not enough; the controller needs forecasts of fatigue accumulation, overload risk, re-entry probability after interruption, premature exit from high engagement, and state-conditioned intervention response. The advantage sought is lead time: drift detected before overt collapse permits lower-burden correction, whereas detection after collapse yields recovery rather than prevention—a materially weaker claim.

### 8.4 Layer 4: closed-loop intervention

The controller selects *u*_*t*_ according to the belief state, predicted transition, task context, user preference, and safety constraints. Candidate interventions include adaptive pause timing, attention-reset cues, task-framing adjustments, pacing changes, sensory environment modulation, breathing protocols, neurofeedback, task or automation-level reallocation (Parasuraman et al., 2000; Byrne and Parasuraman, 1996), and—where ethically and medically appropriate, and subject to the safety literature and its documented null results (Bikson et al., 2016; Antal et al., 2017; Horvath et al., 2015)—non-invasive neuromodulation.

The central claim is not that any specific intervention is universally effective. It is that intervention effects are *state-dependent and individualized*, and that closed-loop selection is the mechanism that makes personalization meaningful. This claim is precisely the one that a micro-randomized design tests, because a state-dependent effect appears as a moderation of the causal excursion effect by the tailoring variable (Klasnja et al., 2015; Liao et al., 2016).

### 8.5 Layer 5: personal neural twin

The personal neural twin is the longitudinal individualized model *M*_*i*_ = {*f*_*i*_, *g*_*i*_, *J*_*i*_, Π_*i*_}, where *f*_*i*_ models state transitions, *g*_*i*_ the observation process, *J*_*i*_ task-conditioned performance, and Π_*i*_ the mapping from state and context to expected intervention effect. Section 9 specifies it in detail.

### 8.6 Information flow and separation of concerns

Two rules govern the interfaces. Each layer passes *distributions with uncertainty*, not point estimates, since collapsing the posterior early discards exactly the information the safety layer needs. And the twin never acts directly: it supplies parameters to layers 2–4 and receives evidence back, but every action is emitted through the constrained policy and safety layer, keeping the learned, drifting, personalized component behind a fixed, auditable gate.

## 9 The personal neural twin: operational specification

The personal neural twin is the element most at risk of being a vision statement rather than a specified construct. This section sets out what the twin is, how it differs from existing digital-twin and personalized machine-learning work, how it would be built, updated, and validated, and how it fails.

### 9.1 Definition, and what the twin is not

The twin is a constrained predictive model of the variables governing state access and control for one individual. It is not a simulation of a brain, not a model of the person, and not a general-purpose representation of cognition. Its value is measured by prediction accuracy, intervention-selection quality, improvement over population baselines, and compounding longitudinal gain.

The National Academies definition of a digital twin requires that the model be personalized to a specific system, dynamically updated with data from that system, predictive, and used to inform decisions, with bidirectional interaction between the virtual and physical counterparts (National Academies of Sciences, Engineering, and Medicine, 2024). We adopt this definition rather than coining one. A model that receives data but informs no decisions is a *digital shadow*; one personalized but static is a *digital model*; a population of synthetic individuals is a *virtual cohort*. Only the full bidirectional, predictive, decision-informing loop qualifies.

### 9.2 Relation to the digital-twin and personalized-ML literature

This matters because the health digital-twin literature does not meet its own standard. A scoping review of 149 human-digital-twin studies published between 2017 and 2024 found only about 12% fully met the National Academies criteria—substantial fractions being digital shadows, static models, or virtual cohorts—and only two addressed verification, validation, and uncertainty quantification (Katsoulakis et al., 2024). Reviews of medical digital twins reach similar conclusions (Laubenbacher et al., 2024).

Two implications follow. First, the twin specified here is a genuine digital twin under (National Academies of Sciences, Engineering, and Medicine, 2024) only if the loop is actually closed: the model must inform the intervention and be updated by its consequences. Second, on what distinguishes it from ordinary personalized machine learning, the answer is limited and should be stated plainly. Mechanically the twin *is* hierarchical personalized modeling with online updating; there is no novel learning algorithm. What distinguishes it is that the personalized objects are the *transition kernel and intervention-response function of a control loop* rather than a static outcome predictor; that it carries an explicit validation and rollback contract (Sections 9.5, 9.6); and that it is separately falsifiable (Section 9.7). Without those commitments, “personal neural twin” would be a rebranding of personalized modeling and should not be used.

### 9.3 Construction and the cold-start problem

#### 9.3.1 Initialization

A new user begins with *M*_*i*_ from a population prior fitted across prior users, using hierarchical partial pooling so individual parameters shrink toward the population mean in proportion to available evidence (Gelman and Hill, 2007). Where representation learning is used, encoder weights come from cross-user pretraining with per-user alignment (Wu et al., 2022; Congedo et al., 2017).

#### 9.3.2 Characterization

A supervised phase establishes the performance-anchored target region (Section 6.3), requiring concurrent performance measurement across a range of states. This means sampling degraded states through time-on-task, controlled interruption, and controlled pressure manipulation, since spontaneous variation under-represents exactly the regions the controller must recognize.

#### 9.3.3 Data budget

No adequate estimate exists for how much within-person data individualized transition and intervention-response models require, because the studies have not been done. The structure of the answer can be stated. The transition model needs enough observed *departures*, not enough recording time, so the binding quantity is event count: a discrete-time hazard model with a few covariates and person-level random effects will typically need tens of events per person, implying multiple sessions. The intervention-response model Π_*i*_ needs enough randomized decision points to estimate a moderated effect, and that quantity *can* be computed in advance (Liao et al., 2016), as Section 16.4 does. Determining the data budget empirically is itself a legitimate first study and is listed as a short-term milestone in Section 20.

### 9.4 Update schedule

We propose a two-timescale update. Within session, only the filtering state and low-dimensional adaptation parameters update; structural parameters are frozen. Between sessions, the twin re-estimates structural parameters against a replay buffer holding a stratified sample of historical sessions rather than only recent data. Freezing structure within session is a safety property: behavior is stationary and auditable, and a pathological update cannot destabilize an ongoing deployment.

### 9.5 Validation of the twin

The twin is validated on four criteria, in increasing order of stringency.

1. **Prospective forecast accuracy** for state occupancy and time-to-departure, evaluated on *future* sessions, never on held-out samples from the same session, which leaks temporal information.
2. **Calibration**. Predicted probabilities must match observed frequencies, assessed by reliability diagrams, expected calibration error, and Brier score decomposition, with recalibration where required (Guo et al., 2017). A miscalibrated twin is worse than no twin, since the abstention rule consumes the posterior directly.
3. **Decision quality**, by off-policy evaluation of the twin-informed policy against the logged policy with confidence intervals (Levine et al., 2020). Accuracy that does not improve decisions is insufficient.
4. **Superiority over baselines:** a population model without personalization, a per-user model with fixed parameters, and a naive persistence baseline—the last because trivial baselines are frequently competitive in physiological forecasting and frequently omitted.

### 9.6 Failure modes and mitigations

See Table 5 for Failure modes and mitigations.

**Table 5:** Failure modes of the personal neural twin, their observable signatures, and mitigations.

Failure mode	Mechanism	Observable signature	Mitigation
Dataset shift/model drift	Sensor change, seasonal or life change, task change, hardware aging (Finlayson et al., 2021)	Rising calibration error; falling prospective accuracy on recent sessions	Continuous drift monitoring on a held-out recent window; automatic rollback to the population baseline on breach; scheduled recharacterization
Catastrophic forgetting	Sequential updating overwrites earlier regimes, especially rare states (Kirkpatrick et al., 2017; van de Ven et al., 2022)	Performance collapse on previously well-modeled but recently unseen states	Stratified replay buffer; elastic-weight-style regularization toward prior parameters (Kirkpatrick et al., 2017); explicit retention tests on archived sessions
Personalization bias	The twin over-fits the states the user happens to occupy most, and the policy then keeps them there	Narrowing of observed state distribution over time; declining exploration	Persistent randomization at decision points (Klasnja et al., 2015); monitoring of state-occupancy entropy as a system health metric
Self-confirming model	Closed loop generates its own training data, so counterfactuals are never observed (Section 7.6)	High apparent accuracy with poor off-policy value estimates	Micro-randomization; periodic policy-free observation windows
Over-shrinkage	Hierarchical prior dominates a genuinely atypical individual	Individual posterior indistinguishable from the population mean after substantial data	Monitor shrinkage factor per user; report it as a diagnostic
Negative transfer	Population prior drawn from a dissimilar user population	Personalized model underperforms a from-scratch individual model	Source-user selection; alignment before transfer (Wu et al., 2022; Congedo et al., 2017); fairness audit of the prior population (Section 17.4)
Objective misspecification		Users dismiss interventions the system scores as valuable	Periodic preference re-elicitation; dismissal rate as a monitored burden signal
Privacy leakage through the model	Model parameters or outputs reveal sensitive traits	Successful membership or attribute inference on the model	On-device retention of individual parameters; federated aggregation (Rieke et al., 2020); formal privacy accounting where population models are shared

### 9.7 What would falsify the twin

The twin's null hypothesis is specific and testable: **after adequate data exposure, an individualized, longitudinally updated model provides no improvement in prospective forecast calibration or in off-policy decision value over a population-level model with per-session calibration**. If this null cannot be rejected in a longitudinal study of the design specified in Section 16, the fifth layer should be removed from the architecture and the framework reduced to four layers. We state this explicitly because a component that cannot fail is not a scientific proposal.

## 10 Foundation models, agentic AI, and cognitive-state estimation

Recent developments in large-scale pretrained models bear directly on the inference and prediction layers, and a framework proposed now should engage them.

### 10.1 Foundation models for biosignals

Self-supervised pretraining on large heterogeneous EEG corpora has produced general-purpose encoders. LaBraM predicts neural tokens for masked EEG channel patches across roughly 2,500 h of recordings from around 20 datasets (Jiang et al., 2024); later models scale the corpus further (Wang et al., 2025); and earlier work established that pretext tasks on unlabeled clinical EEG recover structure relevant to downstream inference (Banville et al., 2021). The relevance is direct: label scarcity (Section 7.1) is what self-supervised pretraining addresses, and a pretrained encoder feeding a switching state-space model instantiates the hybrid architecture recommended there.

The appraisal must be sober. Independent fine-tuning studies report that current large brainwave models do not consistently outperform well-tuned conventional pipelines, with gains sensitive to evaluation protocol (Lee et al., 2025) —consistent with the finding that deep learning's median advantage over traditional EEG pipelines is real but modest and reproducibility poor (Roy et al., 2019). Foundation models are a promising component of the representation layer, not a solution to state inference, and any system built on them must evaluate against conventional baselines.

### 10.2 Multimodal foundation models and large language models

Two roles are plausible. In *context encoding*, a language model converts unstructured context—calendar, task description, message categories, self-report text—into features for the tailoring variable, never touching physiological signal and never selecting an action. In *explanation generation*, it produces the user-facing account of why an intervention was proposed (Section 17.5), constrained to render the policy's actual reasons rather than invent plausible ones. Using a general-purpose model as the state estimator or controller is not plausible on current evidence: latency, calibration, and the absence of any guarantee that a generated rationale corresponds to the decision process all argue against it, and the cost of a confabulated state estimate is an intervention delivered to a human at the wrong moment.

### 10.3 Reinforcement learning agents and agentic orchestration

The policy layer is naturally a reinforcement learning problem, and the offline RL literature supplies both tools and warnings (Levine et al., 2020; Sutton and Barto, 2018). Agentic orchestration—planning multi-step interventions, scheduling recovery, negotiating with other software on the user's behalf—is a plausible medium-term direction and the main route from single interventions to day-level scheduling. It also expands the system's autonomy and attack surface, and is premature until the causal effects in Section 12 are established.

Digital-twin language converging with agentic AI creates a specific risk: a system described as a twin, marketed as agentic, and evaluated as neither. The corrective is the verification, validation, and uncertainty-quantification standard the digital-twin literature itself identifies as largely missing (National Academies of Sciences, Engineering, and Medicine, 2024; Katsoulakis et al., 2024), which is why Section 9.5 is a hard requirement rather than a recommendation.

## 11 Empirical and methodological challenges to the framework

A contribution that ignores evidence against its own assumptions is optimistic by construction. This section reviews five bodies of evidence challenging the framework; Section 11.6 states what each implies.

### 11.1 Reliability and reproducibility of cognitive and physiological markers

The most serious challenge is reliability. Paradigms producing robust group-level effects can have poor test–retest reliability as individual-difference measures, precisely because the low between-subject variance making an effect robust also makes it a weak instrument for measuring individuals (Hedge et al., 2018). For a framework built on individualized modeling this is not peripheral: the tasks most likely to serve as performance anchors may be unreliable at the level at which it operates.

The associated problem in neural measurement is scale. Brain-wide association studies require thousands of participants for stable estimates, and smaller-sample effects are systematically inflated (Marek et al., 2022); analytic flexibility compounds this, since independent teams analyzing one dataset reach materially different conclusions (Botvinik-Nezer et al., 2020). Explanatory and predictive modeling have distinct standards, and conflating them is a persistent source of over-claiming (Poldrack et al., 2020; Hernán and Robins, 2020).

For physiological markers, non-stationarity across sessions, days, and devices is the normal condition and the principal reason EEG-based systems require recalibration (Wu et al., 2022). Reviews of real-world workload measurement conclude the construct lacks a consensual operational definition and that methods have not converged (Diarra et al., 2025).

### 11.2 Mixed findings in neurofeedback

Neurofeedback is the closest intervention paradigm to Layer 4, and its evidence base is contested: benefits may arise from the process being trained or from non-specific factors: expectancy, environment, practice, and separating them requires sham or active controls much of the literature lacks (Ros et al., 2020; Thibault et al., 2016). The field's response was the CRED-nf checklist, specifying the controls, blinding, and reporting an interpretable claim requires (Ros et al., 2020); we adopt it as mandatory in Section 16.11.

The same caution applies to non-invasive neuromodulation. Quantitative review finds no reliable cognitive effect of single-session transcranial direct current stimulation in healthy populations (Horvath et al., 2015), and while safety parameters are well characterized (Bikson et al., 2016; Antal et al., 2017), efficacy for enhancement is unestablished. Neuromodulation is admissible only where state-conditioned effects meet Section 12 standards; its place in the candidate set is a hypothesis, not an endorsement.

### 11.3 Cross-task and cross-subject generalization

Cognitive-state classifiers generalize poorly across subjects, sessions, devices, and tasks. This is the explicit motivation for the transfer-learning literature in EEG-based BCI, which exists because a generic model optimal across subjects and sessions has proven infeasible and calibration is otherwise required for every new user, session, or device (Wu et al., 2022). Cross-task transfer is the least solved of these settings, and independent evaluation finds that large brainwave models do not consistently outperform conventional pipelines after fine-tuning (Lee et al., 2025). Assumption A3 is the direct response: state structure is defined within a task class, and the framework does not require cross-task transfer to succeed. This is a genuine reduction in scope, stated as such rather than treated as an incidental restriction.

### 11.4 Ecological validity of wearable neurotechnology

Laboratory demonstrations do not transfer straightforwardly to the field. Mobile brain–body imaging has made recording during natural behavior feasible (Makeig et al., 2009; Gramann et al., 2014; Debener et al., 2012) and wearable dry-electrode systems support monitoring outside the laboratory (Mullen et al., 2015), but motion, electrode drift, ambient noise, perspiration, and the mechanics of a working environment degrade signal quality; ear-centered and concealed montages improve wearability at the cost of spatial coverage (Bleichner and Debener, 2017); and few methods have been validated in the field rather than the laboratory (Diarra et al., 2025). A burden paradox is specific to this framework: the sensing required to detect a fragile state may itself perturb that state—not a hypothetical concern for a system whose objective includes a burden term.

### 11.5 Laboratory metrics and real-world performance

The final challenge concerns whether improvements measured on cognitive tasks translate to operational outcomes. The cognitive-training literature is the cautionary case: extensive review finds that brain-training programs improve trained tasks reliably, closely related tasks less consistently, and everyday cognitive performance not at all (Simons et al., 2016); meta-analysis of working-memory training likewise finds no convincing far transfer (Melby-Lervåg et al., 2016).

The framework's structural response is that its endpoints are dynamic properties of state occupancy during the operational task itself, not trained-task scores. It therefore does not claim transfer, because it does not train a capacity and hope it generalizes. It remains exposed to the underlying warning, however—improvements in a laboratory-instrumented task need not appear in the uninstrumented operational version—and Section 16.8 accordingly requires external validation in the deployment setting.

### 11.6 Design consequences

Table 6 states what each challenge implies. These are not caveats appended to the framework but constraints that shaped it.

**Table 6:** Empirical challenges, their consequences for the framework, mitigations adopted, and the corresponding falsifier.

Challenge	Consequence for the framework	Mitigation adopted	Corresponding falsifier
Reliability paradox in cognitive tasks (Hedge et al., 2018)	Performance anchors may be unreliable at the individual level	Select anchors by measured within-person reliability, not by convention; aggregate over many trials; report reliability alongside every anchor	Assumption A2 fails if within-person cross-session identification does not exceed chance
Small-sample instability of brain–behavior association (Marek et al., 2022; Botvinik-Nezer et al., 2020)	Population priors estimated from small cohorts will be unstable	Hierarchical modeling with honest uncertainty; population prior treated as weakly informative; no claim from small-N population effects	Twin null (Section 9.7) is not rejected because the prior carries no usable information
Non-stationarity across sessions and devices (Wu et al., 2022)	Fixed models degrade; recalibration is unavoidable	Two-timescale updating with drift monitoring and rollback (Section 9.4, 9.6)	Drift rate exceeds the rate at which the twin can re-estimate
Non-specific effects in neurofeedback (Ros et al., 2020; Thibault et al., 2016)	Apparent benefit may not be attributable to the loop	Sham and active control conditions; CRED-nf reporting; closed-loop vs. open-loop as the decisive comparison	Claim 6.4 fails if closed-loop does not exceed open-loop and sham
Null cognitive effects of single-session tDCS (Horvath et al., 2015)	Neuromodulation cannot be assumed effective	Neuromodulation admitted only as a hypothesis under Section 12 standards	State-conditioned neuromodulation effects indistinguishable from state-independent effects
Poor cross-task and cross-subject transfer (Wu et al., 2022; Lee et al., 2025)	Task-general state ontology is not available	Assumption A3 restricts scope to within task class; alignment before transfer	Within-task-class structure proves as unstable as cross-task structure
Ecological-validity gap (Mullen et al., 2015; Diarra et al., 2025; Bleichner and Debener, 2017)	Laboratory performance overstates field performance	Field validation required before any deployment claim; burden term in the objective	System that works in-laboratory shows no benefit in the operational setting
Far-transfer failure in cognitive training (Simons et al., 2016; Melby-Lervåg et al., 2016)	Improvement on instrumented tasks may not generalize	Endpoints defined on the operational task; external validation in the deployment setting (Section 16.8)	Benefit does not survive removal of the instrumented context

## 12 Predictive modeling vs. causal control

The framework assumes that state estimation and transition prediction can support interventions which causally improve performance, while much of the literature it draws on supports correlational prediction rather than causal control. This section separates the claim types and states the evidence each requires.

### 12.1 Three distinct claims

- **Associational**. Physiological or behavioral features covary with performance-relevant states. Evidence: observational correlation. This is the claim best supported by existing literature.
- **Predictive**. Given observations up to time *t*, the future state or performance at *t*+Δ can be forecast better than by a baseline, on data never used in fitting. Evidence: prospective out-of-sample forecasting with appropriate temporal validation. Note that predictive validity does not require any causal understanding, and that a purely predictive model gives no warrant for intervening (Bzdok and Ioannidis, 2019; Poldrack et al., 2020; Hernán and Robins, 2020; Boruvka et al., 2018).
- **Causal**. Delivering intervention *u* in state *x* changes the distribution of subsequent states or performance relative to withholding it. Evidence: randomization of *u*, or an equally credible identification strategy.

### 12.2 Mapping the framework's claims

Restating the testable claims of the framework with their type made explicit:

**Claim 1 (Signature existence)—associational and predictive**. For a given individual and task class, high-performing states are associated with recurrent patterns in behavioral, physiological, contextual, and neural signals, and those patterns identify the state on held-out sessions.
**Claim 2 (Predictable drift)—predictive**. Departures from high-performing states are preceded by measurable transition trajectories with lead time exceeding intervention actuation latency, forecast better than persistence and base-rate baselines.
**Claim 3 (Corrective controllability)—causal**. State-conditioned interventions alter transition probabilities favorably, increasing dwell time or reducing recovery latency, beyond sham, generic prompting, expectancy, and ordinary rest.
**Claim 4 (Longitudinal learnability)—predictive and causal**. Individualized models improve forecast calibration and, through improved action selection, decision value, relative to population models.
Only Claims 3 and 4 require causal evidence, and Claim 3 is the load-bearing one: without it, the framework reduces to a monitoring system.

### 12.3 Evidentiary requirements for the causal claims

The framework commits to the following standards, which are also reflected in the validation design.

#### 12.3.1 Randomization at the decision point

The primary design is the micro-randomized trial, in which intervention options are randomized at each of many within-person decision points, permitting estimation of the causal excursion effect and of its moderation by the inferred state (Klasnja et al., 2015; Liao et al., 2016). Moderation by state is the operational form of Claim 3: a state-dependent effect *is* a moderated excursion effect.

#### 12.3.2 Control conditions that isolate the loop

The decisive comparison is not intervention vs. nothing but closed-loop state-conditioned intervention (C3) vs. open-loop intervention matched on dose, timing distribution, and content (C2). This isolates the contribution of state conditioning from the contribution of the intervention itself, and is the design that produced interpretable evidence in adaptive DBS (Oehrn et al., 2024).

#### 12.3.3 Blinding

Participants should be blind to condition assignment where feasible; assessors and analysts should be blind throughout. Where participants cannot be blinded to the presence of an intervention, they can often be blinded to whether its timing is state-conditioned.

#### 12.3.4 Negative controls

Effects should appear on targeted states and not unrelated ones; sham interventions matched for interruption cost but not content test whether the effect is attributable to interruption alone.

#### 12.3.5 Dose–response

Where an intervention has a graded parameter, monotonicity strengthens causal interpretation.

#### 12.3.6 Mediation

Claim 3 asserts the intervention changes the state and the state change improves performance; testing it requires mediation analysis with the inferred state as mediator, preregistered as secondary given the known difficulties of mediation inference.

### 12.4 Threats specific to this design

Regression to the mean is a serious threat because interventions are triggered by extreme values of the state estimate, and extreme values are followed by less extreme ones regardless of action; randomized withholding controls this directly and is the main reason we require it. Expectancy and novelty effects are documented problems here (Ros et al., 2020; Thibault et al., 2016). Ordinary rest is a competing explanation for any benefit of a system whose interventions include pauses, and must appear as an explicit control arm. Finally, the closed loop confounds its own evaluation, which is why persistent randomization and periodic policy-free observation windows are architectural requirements rather than study-design details (Sections 7.6, 9.6).

## 13 Operational definitions and measurement

The framework defines five terms plus one governing concept. Table 7 gives, for each, an operational definition, candidate measurable indicators, an evaluation metric, and the observation that would count against it. Prose definitions follow in condensed form.

**Table 7:** Operational definitions, candidate biomarkers, evaluation metrics, and falsifiers.

Term	Operational definition	Candidate indicators	Evaluation metric	Falsifier
Peak state	Occupancy of the performance-anchored target region $N\left(x_{i,\tau }^{⋆}\right)$, identified as a level set of the fitted state-to-performance mapping (Section 6.3)	High accuracy with low response variability; efficient latency; stable working-memory maintenance; low re-entry cost; reduced error inflation under complexity	Posterior occupancy probability; *T*_*dwell*_	No latent region shows reproducible association with elevated performance within person and task class
Drift	A trajectory whose predicted hazard of leaving $N\left(x_{i,\tau }^{⋆}\right)$ exceeds a preregistered threshold before overt performance loss	Rising response variability; increased re-reading or rework; slower re-entry; gaze dispersion; motor timing variability; physiological stress signatures; effort-inflation self-report	Lead time at fixed false-alarm rate; *D*_*drift*_	Lead-time distribution lies below intervention actuation latency (assumption A4 fails)
Recovery	Return to $N\left(x_{i,\tau }^{⋆}\right)$ after a departure, characterized jointly by latency, completeness, post-return stability, and burden incurred	Resumption lag; variability after return; sustained occupancy after return	*T*_*recovery*_; post-return dwell; burden-adjusted recovery efficiency	Faster recovery is achieved only at burden cost exceeding its benefit
Cognitive noise	Task-irrelevant internal activity that reduces the ratio of task-relevant to total processing, inferred from performance-anchored variance not attributable to task difficulty	Intrusive thought and mind-wandering probes; attention-switching residue; unstable intention maintenance; increased intra-individual variability	Residual response variability at matched difficulty; probe-caught off-task rate	Construct does not separate from fatigue or from task difficulty in factor structure
Pressure-stable cognition	Preservation of target-region occupancy under an experimentally manipulated increase in evaluative, time, or uncertainty pressure	Occupancy retention under pressure manipulation; sympathetic activation with maintained performance	Dwell time under pressure relative to matched no-pressure baseline; pressure level at which occupancy degrades	Occupancy under pressure is not predictable from occupancy without it, and does not respond to intervention
State sovereignty	The degree to which the user, not the system or an institution, determines the target state, the admissible intervention set, and the data retained	Proportion of interventions user-configurable; refusal and override rates honored; user's ability to inspect and correct the inferred state	Audit of override honoring; user-reported control; retention and access logs	Governing metric, not an empirical construct; violated by design rather than falsified by data

**Peak state** is identified functionally, not by subjective feeling. **Drift** is valuable because it is early; drift caught late is not drift but failure. **Recovery** must be measured with burden included, since fast restoration at high fatigue cost may be inferior to a slower low-burden strategy. **Cognitive noise** is not reduced by sedation: the target is improved signal-to-noise with task-relevant throughput preserved. **Pressure-stable cognition** requires an independent pressure manipulation to be identified at all, which is why Section 16.2 requires one rather than relying on naturally occurring variation.

## 14 Practical feasibility and technological constraints

The framework is technically appealing on paper. Current wearable neurotechnology cannot yet implement it in full, and a balanced statement of why is necessary. Table 8 summarizes what is feasible now and what is not at consumer- or field-grade instrumentation.

**Table 8:** Assessment of what is feasible now vs. what is not, at consumer- or field-grade instrumentation.

Capability	Current feasibility	Principal limiting factor
Detection of gross arousal and drowsiness changes	Feasible	Already demonstrated in field settings (Borghini et al., 2014)
Detection of coarse workload changes within a session and task	Feasible with per-session calibration	Cross-session drift (Wu et al., 2022)
Task-telemetry-based drift detection	Feasible	Requires instrumented task
Within-person, cross-session identification of a performance-anchored target region	Plausible but unproven	Reliability and non-stationarity (Hedge et al., 2018; Wu et al., 2022)
Drift forecasting with minutes-scale lead time	Unproven	Forecast horizon vs. signal informativeness (Section 14.3)
Demonstrated causal benefit of state conditioning over matched open-loop	Unproven in cognitive domains; demonstrated in adaptive DBS (Oehrn et al., 2024)	Requires the design in Section 16
Longitudinal individualized twin outperforming population models	Unproven	No adequate longitudinal datasets exist
Fine-grained discrimination among adjacent states such as flow vs. deep focus	Not currently feasible	Constructs themselves not established (Alameda et al., 2022)
Non-invasive neuromodulation as a reliable state-shifting actuator	Not established	Null findings in healthy populations (Horvath et al., 2015)

### 14.1 Signal quality

Dry-electrode and consumer-grade EEG have lower signal-to-noise ratios and higher artifact rates than laboratory systems, and the gap widens under movement (Mullen et al., 2015; Bleichner and Debener, 2017). Optical measures are affected by ambient light and tissue optical properties; autonomic measures are confounded by activity, posture, thermoregulation, caffeine, and medication. None is fatal, but each reduces the effective information rate below what laboratory results imply.

### 14.2 Sensor fusion and missingness

Streams arrive at different rates and drop out independently, so fusion needs an explicit temporal model with per-channel reliability weighting, and the estimate must degrade gracefully to the remaining channels. As in Section 8.1, missingness is informative and must be modeled rather than imputed away.

### 14.3 Latency budget

End-to-end latency determines whether prevention is possible. A realistic budget is: acquisition and buffering 50–250 ms; inference 50–200 ms; forecast 10–100 ms; policy and safety check under 50 ms; delivery to onset of effect 0.5–5 s for a cue or pacing change, and considerably longer for a breathing protocol or break. The binding constraint is therefore not computation but the intervention's own time constant. Since Claim 2 requires lead time exceeding this total, an intervention needing 2 min to take effect requires drift prediction at a 2-min horizon—a far harder forecasting problem than the second-scale problem the sensing literature usually addresses. This **horizon–potency trade-off** is, in our view, the most underappreciated engineering constraint in this class of system: the more potent the intervention, the harder the prediction it demands.

### 14.4 Calibration, compliance, and environment

Every deployment faces the calibration burden motivating the transfer-learning literature (Wu et al., 2022); since users will not tolerate long calibration sessions, the practical path is population priors with rapid alignment (Section 7.4) and characterization spread across ordinary use. Adherence to wearable protocols degrades over weeks, and notification fatigue is well documented (Nahum-Shani et al., 2018): a system that intervenes too often is disabled by its user—the most common failure mode of deployed adaptive systems, and rarely reported as a scientific result. Temperature, humidity, vibration, electromagnetic interference, and protective equipment all bear on feasibility in exactly the operational settings where the framework would be most valuable.

## 15 Application scenarios

One scenario is developed in full to make the loop timing explicit; a second is proposed as the first research setting; other domains are treated briefly, each being dominated by a governance rather than technical constraint.

### 15.1 Long-haul flight deck (worked example)

#### 15.1.1 Setting

Cruise phase of a long-haul flight: low task demand, high consequence, well-instrumented cockpit. Vigilance decrement and sleep-related attentional lapses are the dominant risk and among the best-characterized phenomena in the literature (Warm et al., 2008; Lim and Dinges, 2008; Borghini et al., 2014).

#### 15.1.2 Sensing

Ear-centered or headset-integrated EEG (Bleichner and Debener, 2017), eye tracking already present for other purposes, cardiac data from a wrist device, and flight-management-system telemetry giving response latency to system prompts and interaction cadence.

#### 15.1.3 Characterization

The target region is anchored to detection latency and false-response rate on routine system interactions, established over preceding flights rather than a dedicated session.

#### 15.1.4 The loop

A hazard model estimates the probability of leaving the target region within the next 10 min, conditioned on time on task, prior sleep, phase of flight, and current belief state. When the hazard crosses a preregistered threshold, and the safety layer confirms that the posterior is not diffuse and the burden budget is not exhausted, the policy selects among a small admissible set: a task-relevant verification prompt, a scheduled controlled rest under existing crew procedures, or a lighting or auditory environment change.

#### 15.1.5 Timing

The forecast horizon is 10 min, well above the actuation latency of any of these interventions—which is why this setting is a plausible first target: the intervention time constants are long, but so is the phenomenon predicted.

#### 15.1.6 Evaluation

Endpoints are dwell time in the target region, lead time before objectively detected lapses, and the rate of prompted vs. spontaneous detection of system events—all obtainable in high-fidelity simulation before airborne evaluation.

#### 15.1.7 Dominant constraint

Certification and the regulatory status of any system influencing flight-crew behavior, together with the requirement that the system never substitute for crew-rest regulation.

### 15.2 Complex knowledge work (recommended first research setting)

Software development, analysis, and writing are attractive early settings because task telemetry is rich and free, interruption is the dominant degradation, the intervention set is broad and low-risk, and ethical stakes are lowest for consenting adult volunteers using the system on their own work. With the flight-deck simulator case, we regard this as the appropriate setting for a first prototype (Section 20).

### 15.3 Other domains

Three further domains are frequently proposed and each is dominated by a non-technical constraint. In **surgery**, instrument tracking provides precise telemetry, but real-time intervention during a critical maneuver is indefensible on burden and interruption grounds; the defensible role is *scheduling*—deciding whether the next elective case should be delayed or reassigned—and the dominant constraint is the employment and liability exposure created by inferring a clinician's state. In **long-haul driving**, coarse drowsiness detection is already deployed and the framework would add earlier, individualized drift detection; the dominant constraint is that the most effective intervention, stopping, carries an economic cost that creates structural pressure to under-trigger, which is why the burden term must be user- or safety-anchored rather than operator-anchored. **Education** is the most frequently proposed and the least defensible near term: learners are often minors, the power asymmetry is severe, inferring emotional state from biometric data in educational institutions is prohibited in the European Union under Article 5(1)(f) of the AI Act subject to narrow medical and safety exceptions (European Parliament and Council, 2024), and UNESCO advises against non-therapeutic neurotechnology use in children and young people (UNESCO, 2025). We therefore list education as contingent on a regulatory pathway that does not currently exist.

## 16 Materials and methods for future validation

No human participants, animal subjects, empirical measurements, or simulations are reported here. This section specifies a validation program in the detail required to preregister it.

### 16.1 Design

The program follows the optimization-then-evaluation logic of the multiphase optimization strategy, tuning components before a confirmatory trial (Collins et al., 2007). The core design is a within-person randomized comparison of four counterbalanced conditions, with micro-randomization nested within the intervention conditions:

- **C1** unassisted baseline;
- **C2** open-loop intervention, matched to C3 on dose, content, and the marginal distribution of timing, but not state-conditioned;
- **C3** closed-loop state-conditioned intervention using a population model with per-session calibration;
- **C4** closed-loop intervention with longitudinal personalization through the personal neural twin;
- **C5** closed-loop intervention driven by task telemetry and behavioral signals alone, with no physiological or neural sensing.

C1 vs. C3 tests whether the system helps. **C2 vs. C3 is the decisive comparison**, isolating the contribution of state conditioning from the intervention itself. C3 vs. C4 tests the twin (Section 9.7). **C5 vs. C3 tests whether neurophysiological sensing earns its cost**. Behavioral telemetry is free, unobtrusive, individually calibrated by construction, and continuously available (Section 8.1), making it the correct floor against which added sensing must justify burden, price, and privacy exposure. If the incremental value of physiological and neural channels over C5 is small, the defensible product is a telemetry-only system. Within C2 and C3, delivery is micro-randomized at each decision point, enabling estimation of causal excursion effects and their moderation by inferred state (Klasnja et al., 2015; Liao et al., 2016), and enabling off-policy evaluation of candidate policies without deploying them (Levine et al., 2020).

### 16.2 Tasks

Tasks should span sustained attention, working-memory stress, interruption-recovery, complex reasoning, and time-constrained decision making, plus a domain-representative task. At least one must include an explicit, calibrated **pressure manipulation** — evaluative, time, or uncertainty—since pressure-stable cognition cannot otherwise be identified (Table 7). Given the reliability paradox (Hedge et al., 2018), task selection must be justified by measured within-person test–retest reliability of the primary performance measure, and anchors with poor individual-level reliability excluded even when conventional.

### 16.3 Endpoints

*Primary:* entry latency; dwell time; drift-detection lead time at a fixed false-alarm rate; recovery latency after interruption; and a per-task composite performance measure. *Secondary:* accuracy, response latency and variability, error inflation under pressure, intervention burden, subjective effort and clarity, task friction, trust, interruption cost, day-to-day reproducibility, adherence, and adverse events. Subjective measures are secondary because perceived clarity without objective improvement is insufficient, though necessary for usability and safety.

### 16.4 Sample size and statistical power

Three distinct power questions arise, and they have different answers.

#### 16.4.1 Micro-randomized within-person effects

Sample size follows from decision points per person, randomization probability, expected availability, and standardized proximal effect (Liao et al., 2016). Indicatively, a constant standardized proximal effect of 0.10 with 200 decision points per participant, randomization probability 0.5, availability 0.8, at 80% power and two-sided α = 0.05, requires participants on the order of tens rather than hundreds; detecting *moderation* by inferred state—what Claim 3 requires—typically demands a two- to four-fold increase. Exact values must be preregistered under the study's own assumptions.

#### 16.4.2 Between-condition comparison

Because observations are nested within decision points, sessions, tasks, and individuals, power for the C2-vs.-C3 contrast should be established by simulation from a fitted mixed-effects model rather than closed-form formulae assuming independence. Pilot-derived effect sizes should not seed the calculation, since small-pilot effects are upward-biased.

#### 16.4.3 Individual-level model estimation

The binding quantity is the number of observed *departures* per person, not recording hours (Section 9.3). Studies should report events per person and per parameter, and treat an inadequate event count as a reason to extend collection rather than to fit the model anyway.

### 16.5 Participant and dataset diversity

Generalization claims require a sample that supports them. Recruitment should be stratified and reported across age, sex, chronotype, and shift-work status, since baseline and diurnal variation in the target signals differ across them. Two sensing-specific requirements deserve statement. **Skin pigmentation affects optical measurement**: racial bias in pulse oximetry is documented (Sjoding et al., 2020), and any system relying on photoplethysmography or near-infrared measures must validate accuracy across skin tones rather than assume it. **Hair texture and protective hairstyles affect scalp EEG electrode contact**, degrading signal quality with standard montages; studies should report data quality per participant and never silently exclude for “poor signal” without analyzing who is excluded. Neurodivergent participants and users of common psychoactive medications should be included by default, since exclusion produces a model that fails precisely for those who might benefit most. Multi-site recruitment is required for any claim beyond one laboratory.

### 16.6 Longitudinal follow-up

Single-session experiments cannot test the framework's strongest claim. A longitudinal protocol should span several weeks at multiple sessions per week, enough to accumulate the event counts in Section 16.4 and observe at least one natural perturbation such as sleep restriction. Follow-up must extend beyond the intervention period to detect whether benefits persist, decay, or reverse, and to detect dependence: a system that improves performance while worn and degrades unassisted performance afterwards is a harm, and only follow-up reveals it.

### 16.7 Model calibration and uncertainty

Calibration is a primary model property, because the safety layer consumes posterior probabilities. Studies should report reliability diagrams, expected calibration error, and Brier score with decomposition before and after any recalibration (Guo et al., 2017), quantify predictive uncertainty by the method appropriate to the model family (Gal and Ghahramani, 2016), and report calibration *prospectively on future sessions* rather than on held-out samples from the same session.

### 16.8 External validation and generalization

Internal validation is insufficient for any deployment claim. The program requires temporal validation on later sessions, cross-site validation with different hardware and personnel, and validation in the uninstrumented operational setting, addressing the far-transfer concern in Section 11.5. Performance must be reported separately for each pre-specified subgroup, never pooled away.

### 16.9 Blinding, sham, and adverse events

Assessors and analysts blind throughout; participants blind to state-conditioning where feasible. Include a sham condition matched for interruption cost but not content. Define adverse events in advance and collect them actively—sleep disruption, anxiety, compulsive use, over-reliance—reporting them irrespective of attribution.

### 16.10 Analysis

Preregister primary endpoints, the minimum operationally meaningful difference, exclusion criteria, and the analysis plan before collection. Mixed-effects models suit the nesting structure. Cross-validation must be across days and tasks, never across adjacent samples within a session, which leaks autocorrelation and inflates apparent performance. Causal effects require methods appropriate to time-varying treatments under time-varying confounding (Klasnja et al., 2015; Boruvka et al., 2018).

### 16.11 Reporting standards

The program should conform to CRED-nf for any neurofeedback component (Ros et al., 2020), TRIPOD+AI for the prediction models (Collins et al., 2024), and SPIRIT-AI and CONSORT-AI for protocols and trial reports (Liu et al., 2020; Cruz Rivera et al., 2020). The responsible-machine-learning-for-health literature adds obligations across the model lifecycle, including problem selection, deployment monitoring, and accountability (Wiens et al., 2019; Char et al., 2018). Committing to these in advance is the best available protection against the reporting problems in Section 11.

## 17 Safety, ethics, and governance

A cognitive-state control system raises safety and governance issues even when interventions are non-invasive.

### 17.1 Agency, consent, and reversibility

Systematic surveys of human-computer interface ethics in assistive systems converge on the obligations addressed here—autonomy, privacy, fairness, explainability, consent, and accountability for adaptive behavior—and find them enumerated far more often than operationalized (Ejaz et al., 2026). The commitments below are therefore stated as design requirements.

Interventions must be consent-based, interruptible, reversible, and transparent. The user must be able to inspect the inferred state, see why an intervention is proposed, and refuse without penalty. Override must be honored architecturally rather than as a preference setting, and the learning system must not treat refusal as a cue to intervene differently at the same moment.

### 17.2 Mental privacy and neural data

Neural, behavioral, and physiological signals can reveal fatigue, stress, attention, affect, health conditions, and performance vulnerabilities. Data minimization, on-device processing, encryption, access control, and strict retention limits are core design requirements rather than compliance afterthoughts. Federated approaches allow population-level improvement without centralizing raw neural data (Rieke et al., 2020), though they do not by themselves prevent inference from model updates.

An international standard now supports this position: UNESCO's Recommendation on the Ethics of Neurotechnology, adopted in November 2025, treats neural data as uniquely sensitive, requires safeguards against misuse, protects freedom of thought and the right not to be subject to neurotechnology, and warns specifically against workplace use to monitor productivity or build employee profiles (UNESCO, 2025). Earlier scholarship anticipated this framing in proposing mental privacy and cognitive liberty as rights claims (Ienca and Andorno, 2017; Yuste et al., 2017).

### 17.3 Cybersecurity of the closed loop

A closed-loop system has an attack surface a monitoring system does not, since compromise leads to action rather than only disclosure. The threat model includes exfiltration of neural data; sensor spoofing to induce a false state estimate; poisoning of longitudinal training data so the twin learns a harmful policy; adversarial perturbation at inference; compromise of the intervention channel; and inference attacks recovering sensitive attributes from model parameters. Mitigations include authenticated sensor channels, plausibility checks on physiological inputs, rate limiting and refractory periods enforced in the non-learned safety layer, anomaly detection on the update stream, signed model versions with rollback, and an append-only audit log. The rule in Section 8.6—the twin never acts directly—exists partly for this reason.

### 17.4 Algorithmic bias and fairness

Bias enters by four routes: unrepresentative population priors; sensing with unequal accuracy across groups, as documented for optical measures (Sjoding et al., 2020) and as arises for scalp EEG across hair textures; performance anchors encoding one working style as optimal; and differential exclusion, in which participants with noisier signals are removed from datasets and therefore from the models. The result is a system working least well for groups already least well served, a pattern documented in health algorithms generally (Obermeyer et al., 2019). Mitigations must be specified in advance: subgroup-stratified reporting of accuracy and calibration; fairness constraints or reweighting where disparities appear; documentation of the prior population; and a rule that materially degraded subgroup performance is a blocking finding, not a limitation to be noted.

### 17.5 Explainability of intervention decisions

A user asked to accept a real-time intervention is entitled to know why. Three levels are appropriate: the inferred state and its confidence; which feature or forecast crossed which threshold; and what would have prevented the action. Because the explanation shapes willingness to comply, one that is persuasive but unfaithful is a manipulation risk rather than an accuracy failure—an argument for interpretable models in the decision path wherever performance permits, rather than *post-hoc* explanation of an opaque model (Rudin, 2019). Where a learned component is retained, explanations must derive from the policy's actual inputs and thresholds, never be generated freely by a language model (Section 10.2).

### 17.6 Informed consent for adaptive, learning systems

Consent for a system whose behavior changes with accumulated data cannot be a single event: what the user agrees to at enrollment is not what the system does 6 months later. Requirements are consent to the *class* of interventions and data collected, with per-modality opt-out; notification when the model updates materially or a new intervention type activates; the ability to inspect, export, and delete the personal model and its training data, including a right to reset the twin to the population baseline; and re-consent at defined intervals. Where an employer or school obtains consent, it is presumptively compromised by the power asymmetry—the substantive reason behind the regulatory positions below.

### 17.7 Coercive deployment, workplace, and educational settings

Cognitive-state technologies can be misused in workplaces, schools, military settings, and competitive environments. A platform designed for state sovereignty is ethically inverted if institutions use it to extract longer work periods without respecting autonomy, recovery, and refusal.

In some jurisdictions this is now a legal constraint. Article 5(1)(f) of Regulation (EU) 2024/1689 prohibits placing on the market, putting into service, or using AI systems to infer emotions of natural persons in workplaces and educational institutions, with narrow medical and safety exceptions; it has applied since 2 February 2025 (European Parliament and Council, 2024). A system inferring affective interference or stress from physiological signals at work falls within the territory it governs, and the boundary between prohibited emotion inference and permitted safety-motivated fatigue monitoring is the question a deployment must resolve with counsel before, not after, development. UNESCO adds non-binding guidance against workplace productivity monitoring and non-therapeutic use in children (UNESCO, 2025). Development programs should therefore treat safety-critical operator monitoring with an explicit safety justification as the near-term regulatory path, and productivity-motivated deployment as out of scope.

### 17.8 Regulatory pathway and clinical claims

A performance platform must not be marketed as treatment for attention-deficit/hyperactivity disorder, anxiety, depression, fatigue syndromes, traumatic brain injury, or other clinical conditions without trials and regulatory review. Classification as a general-wellness product rather than a medical device turns on claims and risk: a system that stops describing itself as supporting focus and starts describing itself as treating a condition changes category (U.S. Food Drug Administration, 2019). Safety-critical settings add sector-specific oversight, and classification should be settled before design freeze, since the required evidence differs by an order of magnitude between paths.

### 17.9 Individualized safety

A state target that improves productivity while increasing stress, sleep disruption, anxiety, compulsive overwork, or affective instability is not acceptable. The feasible set *F*_*i*, τ_ must include health and wellbeing constraints, enforced in the safety layer rather than expressed as objective weights—weights can be traded off, constraints cannot.

## 18 Discussion

The framework has two prospective strengths, neither demonstrated: it narrows a vague enhancement idea into measurable variables, and identifies closed-loop personalization as the central mechanism. The question becomes tractable: whether target states can be inferred within person and task class, whether drift can be predicted with usable lead time, and whether state-conditioned interventions alter trajectories at acceptable burden.

The risks are correspondingly sharp. Latent states may be less stable than the model assumes, and the reliability literature gives real grounds for that worry (Hedge et al., 2018; Marek et al., 2022). Labels may fail to transfer across tasks, which is why the framework retreats to within-task-class operation (Wu et al., 2022; Lee et al., 2025). Gains may be explained by placebo, motivation, novelty, or ordinary rest, which is why the decisive comparison is C2 vs. C3 (Ros et al., 2020; Thibault et al., 2016). Interventions may create more interruption burden than they relieve, sensing is noisy in everyday settings (Mullen et al., 2015; Diarra et al., 2025), and laboratory results may not survive uncontrolled environments (Simons et al., 2016; Melby-Lervåg et al., 2016). These risks are the substance of the problem, not caveats on it.

Two constraints seem underappreciated. The **horizon–potency trade-off** (Section 14) means the interventions most likely to work demand the hardest prediction problem. The **self-confirmation problem** (Sections 7.6, 9.6) means a closed-loop system generates the data by which it is evaluated, so persistent randomization is architecturally required for it to remain evaluable at all.

No simulation is offered in place of evidence, because a toy validator with synthetic data creates a false sense of it. Nothing here is evidence that the architecture works: no layer implemented, no loop closed, no endpoint measured. Rigor means no efficacy claim without human data, no clinical implication without clinical evidence, and no assertion of state control without a closed-loop vs. open-loop comparison.

The most promising near-term path is not a full cognitive-control system but a narrow, task-conditioned prototype: one that estimates fatigue and attentional drift during a defined task and compares closed-loop against matched open-loop support over repeated sessions, with micro-randomized delivery so causal effects are estimable. If the personal model compounds across days and improves dwell time or recovery latency without excessive burden, the core thesis gains support. If it does not, the framework should be revised or rejected.

## 19 Limitations

This manuscript is conceptual. It presents no experimental data, device performance, software, clinical outcomes, or user studies. The mathematical formulation is a scaffold for future experiments rather than a validated model of neurocognitive dynamics, and the computational methods in Section 7 are candidate families with known properties, not an implementation that has been shown to work on this problem. The term *highest available cognitive state* is now operationally defined (Section 6.3) but is not yet empirically parameterized for any individual or task.

The framework is limited by measurement uncertainty. Non-invasive signals may not carry enough information to distinguish nearby cognitive states in real time. Wearable sensing is more practical but less precise. Implantable sensing is more informative but ethically and clinically inappropriate for enhancement applications. These trade-offs must be resolved empirically.

Three limitations deserve emphasis. The twin's data budget cannot be specified from the literature (Section 9.3); it is an empirical unknown that milestone M2 is designed to determine. Of assumptions A1–A5, A4—informative precursors with usable lead time—remains untested at the horizons potent interventions require. And the novelty is modest and specific: as Section 4.2 states, the components are not new, and without the commitments listed there what remained would be a recombination of existing work.

Finally, the model emphasizes individual optimization. Social, educational, and organizational causes of underperformance should not be reduced to individual state control. A better interface may help a person recover from interruption, but it does not remove the organizational systems that generate unnecessary interruption, and a technology that makes individuals more resilient to poor working conditions can reduce the pressure to improve those conditions. That is a real risk of this class of system and not one the system can solve.

## 20 Research roadmap

Table 9 gives a staged program in which each stage carries a decisive question and a stop rule. A roadmap without stop rules is a plan to continue regardless of results.

**Table 9:** Staged research roadmap with decisive questions and stop rules.

Horizon	Milestone	Decisive question	Minimum viable study	Stop rule
Short (0–18 months)	M1. Within-person state identification	Can a performance-anchored target region be identified within person and task class, and re-identified on future sessions?	Single task class, 20–40 participants, 8–12 sessions each, laboratory or instrumented knowledge work; report within-person cross-session identification and its reliability	Stop if cross-session identification does not exceed chance after adequate data (A2 fails)
Short	M2. Data-budget characterization	How many sessions and how many observed departures are needed for stable individual transition-model estimates?	Learning curves for individual model parameters as a function of accumulated events, using M1 data	Stop if required event counts are unattainable in realistic use
Short	M3. Drift lead-time characterization	What is the achievable distribution of lead time at operationally acceptable false-alarm rates?	Prospective forecasting on M1 data with hazard and sequence models; report lead time against actuation latency	Stop if lead time is below the actuation latency of every admissible intervention (A4 fails)
Medium (18–36 months)	M4. Causal effect of state conditioning	Does closed-loop delivery outperform matched open-loop delivery?	Micro-randomized trial within C2 and C3, powered for moderation by inferred state per Section 16.4	Stop if the state-moderated excursion effect is indistinguishable from zero (Claim 3 fails)
Medium	M5. Burden-adjusted benefit	Does benefit survive burden accounting and sham control?	Add sham and rest control arms; preregister burden ceiling	Stop if benefit does not exceed sham and rest, or if the burden ceiling is breached
Medium	M6. Twin superiority	Does longitudinal personalization beat a population model with per-session calibration?	C3 vs. C4 over several weeks, with prospective calibration and off-policy value estimation	Remove Layer 5 and reduce the architecture to four layers if the twin null is not rejected (Section 9.7)
Long (3–5 years)	M7. Field and cross-site validation	Does benefit persist outside the instrumented laboratory and across sites, hardware, and populations?	Multi-site deployment in one operational domain with subgroup-stratified reporting	Stop deployment claims if benefit does not survive external validation or if any subgroup shows material degradation
Long	M8. Durability and dependence	Do benefits persist, decay, or reverse after withdrawal?	Extended follow-up beyond the intervention period	Halt if unassisted performance degrades post-withdrawal
Long	M9. Governance and regulatory qualification	Can the system be deployed within the applicable regulatory framework with a defensible safety justification?	Classification determination; conformity work; independent ethics and security review	Do not deploy in workplace or educational settings absent a lawful basis (Section 17.7)

Stages M1–M3 are observational and forecasting studies requiring no intervention, and are therefore both the cheapest and the most likely to falsify the framework early. We regard that ordering as a feature.

## 21 Conclusions

Closed-loop cognitive state control—informally, supercognition—is best understood not as intelligence amplification but as a control objective: reliable access to the highest available task-specific cognitive state, with stabilization and recovery under fatigue, distraction, overload, and pressure.

The framework treats cognition as a partially observable dynamical system. It defines target states, drift, recovery, cognitive noise, and pressure stability operationally; proposes an optimization objective with an account of how it would be estimated; specifies candidate implementations for each layer; and gives a five-layer architecture in which the personal neural twin is a separately falsifiable component rather than an aspiration. It states its assumptions as five testable propositions, separates predictive from causal claims, and engages the literature challenging its feasibility rather than only the literature supporting it. None of this is evidence that the architecture performs as described: the validation programme is entirely prospective, and every claim about system behavior here is a hypothesis awaiting that test.

The ordering of the research program matters: the observational and forecasting milestones that could falsify the framework are also the cheapest to run. The key question is whether individualized closed-loop state control improves entry, dwell, drift prevention, and recovery more than unassisted baseline or matched open-loop assistance, at burden the user accepts. If it does, the most useful near-term form of cognitive enhancement may not be raising trait intelligence but making high-functioning cognition reliably accessible when it matters. If not, the framework has been specified precisely enough to be abandoned.

## Data Availability

The original contributions presented in the study are included in the article/supplementary material, further inquiries can be directed to the corresponding author.
